# Mouse model of atypical DAT deficiency syndrome uncovers dopamine dysfunction associated with parkinsonism and ADHD

**DOI:** 10.1172/JCI169297

**Published:** 2026-01-27

**Authors:** Freja Herborg, Lisa K. Konrad, Søren H. Jørgensen, Jamila H. Lilja, Benoît Delignat-Lavaud, Leonie P. Posselt, Ciara F. Pugh, Sofie A. Bach, Cecilia F. Ratner, Nora Awadallah, Jose A. Pino, Frida Berlin, Aske L. Ejdrup, Mikkel V. Olesen, Mattias Rickhag, Birgitte Holst, Susana Aznar, Felix P. Mayer, David Woldbye, Gonzalo Torres, Louis-Eric Trudeau, Ulrik Gether

**Affiliations:** 1 Molecular Neuropharmacology and Genetics Laboratory, Department of Neuroscience, Faculty of Health and Medical Sciences, University of Copenhagen, Copenhagen, Denmark.; 2 CNS Research Group, Department of Pharmacology and Physiology, Department of Neurosciences, Faculty of Medicine, Université de Montréal, Montréal, Quebec, Canada.; 3 Centre for Neuroscience and Stereology, Copenhagen University Hospital Bispebjerg-Frederiksberg, Copenhagen, Denmark.; 4 Department of Biomedical Sciences, University of Copenhagen, Copenhagen, Denmark.; 5 Department of Molecular, Cellular, and Biomedical Sciences, City University of New York School of Medicine, New York, New York, USA.; 6 Laboratorio de Bioquímica y Farmacología Molecular, Escuela de Ciencias, Facultad de Ciencias de la Vida, Universidad Viña del Mar, Valparaíso, Chile.; 7 Danish Research Centre for Magnetic Resonance (DRCMR), Department of Radiology and Nuclear Medicine, Copenhagen University Hospital-Amager and Hvidovre, Copenhagen, Denmark.; 8 Department of Molecular Pharmacology and Neuroscience, Stritch School of Medicine, Loyola University, Chicago, USA.

**Keywords:** Genetics, Neuroscience, Mouse models, Movement disorders, Psychiatric diseases

## Abstract

Atypical dopamine transporter (DAT) deficiency syndrome (DTDS) arises from genetic disruption of DAT function and is characterized by early-onset parkinsonism alongside comorbid psychiatric symptoms. However, the underlying pathobiological processes are largely unknown. Here, we present a mouse model of atypical DTDS based on the patient-derived compound heterozygote genotype, DAT-I312F/D421N^+/+^. DAT-I312F/D421N^+/+^ mice exhibited markedly impaired DAT function, leading to widespread changes in dopamine homeostasis, including elevated extracellular dopamine levels, reduced tyrosine hydroxylase and dopamine D1/D2 receptor expression, and decreased evoked dopamine release, mechanistically linked to enhanced tonic D2 autoreceptor inhibition. Fiber photometry measurements revealed disrupted fast striatal dopamine release dynamics, while confocal imaging showed reduced striatal dopaminergic axon fiber density. These neurochemical changes were accompanied by a psychomotor phenotype characterized by hyperlocomotion, enhanced exploration, and pronounced clasping. Both amphetamine and anticholinergic treatment ameliorated the aberrant hyperactivity. Notably, amphetamine-induced dopamine release was profoundly blunted in ventral striatum but largely preserved in dorsal striatum, implicating region-specific dopamine release dynamics as a determinant of divergent behavioral and pharmacological responses. Summarized, our findings uncover multiscale dopamine dysfunction that links presynaptic DAT impairment to synaptic and circuit-level disruptions, offering insight into atypical DTDS and the co-occurrence of movement and psychiatric features.

## Abstract 2

In a new mouse disease model, we explore the behavioral consequences and dopaminergic dysfunction that arise from patient-derived mutations in the dopamine transporter associated with parkinsonism and co-morbid neuropsychiatric disease

## Graphical abstract

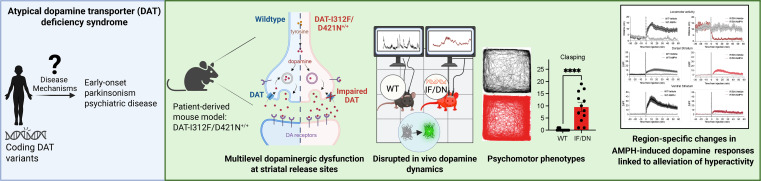

## Introduction

Neuropsychiatric and neurodegenerative diseases of the brain are notoriously difficult to treat, resulting in a large unmet need for developing new treatment strategies. Despite differences in clinical presentation and disease onset, accumulating evidence points to the existence of overlapping pathological mechanisms. For instance, the monoaminergic circuits that show high vulnerability in Parkinson’s disease (PD) (1, 2) have been heavily implicated in the pathophysiology of psychiatric diseases (3–7). There is also evidence that neuropsychiatric disorders and neurodegenerative diseases have mutual risk factors. More than 25% of patients diagnosed with neurodegenerative disorders already have a psychiatric diagnosis, and high rates of psychiatric symptoms have been reported for some genetic forms of PD (8–12). This comorbidity could reflect shared or overlapping etiological factors or commonalities in the molecular and cellular pathways. These factors highlight the need for disease models with construct validity to investigate complex pathobiological processes.

Dopamine (DA) is recognized for its dual role in basal locomotor control and in regulating emotional states that are critical for higher order brain functions, such as attention, motivation, and reinforcement learning. Consistently, DA dysfunction has been implicated in neurodegenerative diseases, particularly PD, and in neuropsychiatric disorders, such as ADHD, bipolar disease, schizophrenia, and substance use disorder (3–6, 13). Indeed, core symptoms of these diseases may be alleviated by therapeutics targeting the DA circuits. However, we still do not fully understand how DA exerts its multiple functions and the nature and progression of DA dysfunction in diseased states. Identifying molecular and neural consequences of DA dysfunction, particularly in relation to behavioral phenotypes, may provide critical insights into disease pathology and thereby support the development of novel pharmacotherapies.

DA homeostasis relies heavily on reuptake via the DA transporter (DAT), which is selectively expressed by DA neurons. DAT thereby ensures a tight spatiotemporal regulation of extracellular DA levels and a synthesis-independent DA supply to dopaminergic terminals (14, 15). The critical function of DAT was underlined when complete loss-of-function mutations in DAT were shown to cause hereditable infantile parkinsonism-dystonia or dopamine transporter deficiency syndrome (DTDS) (16). Moreover, studies of patients with neuropsychiatric disease that carry single-allele coding variants of DAT have provided compelling evidence for a possible causal link between genetic impairments in DAT function and the development of neuropsychiatric diseases (17–22). As a missing link in this emerging DAT-associated disease spectrum, we recently presented the first adult patient with *atypical* DTDS characterized by adult early-onset parkinsonism symptoms and comorbid ADHD. This patient was compound heterozygous for 2 missense DAT variants, DAT-I312F and DAT-D421N, causing a partial loss of DA uptake function in vitro (23). We have since described an additional patient with comorbid parkinsonism and neuropsychiatric disease who carried the dominant-negative DAT-K619N mutation (24). Collectively, the data highlight the importance of understanding the molecular, cellular, and circuitry substrates that link DAT-dependent DA dysfunction to both movement disorders and psychiatric disease.

Here, we generate a construct-valid mouse model of atypical DTDS to elucidate behavioral and neurochemical changes that arise from DAT mutations associated with comorbid early-onset parkinsonism and ADHD. Such a model of highly penetrant disease mutations in key components of the DA system may deepen our understanding of the mechanisms through which DA exerts its multidimensional functions and uncovers common neural substrates for the spectrum of clinical phenotypes shared by atypical DTDS, movement disorders, and neuropsychiatric diseases. The mouse replicates the patient-derived heterozygous DAT-I312F/DAT-D421N genotype (23) and displays a phenotype with prominent changes in DA release and uptake dynamics together with extensive alterations in pre- and postsynaptic components of DA neurotransmission and a reduction in striatal dopaminergic axon fiber density. These changes are accompanied by a behavioral phenotype recapitulating core symptoms of the patient. Collectively, our work presents insights into the neural mechanisms underlying atypical DTDS and points to potential mechanisms underlying comorbidity between neuropsychiatric diseases and movement disorders.

## Results

### DAT-I312F/D421N^+/+^ mice display changes in DA regulation.

**Figure 1:**
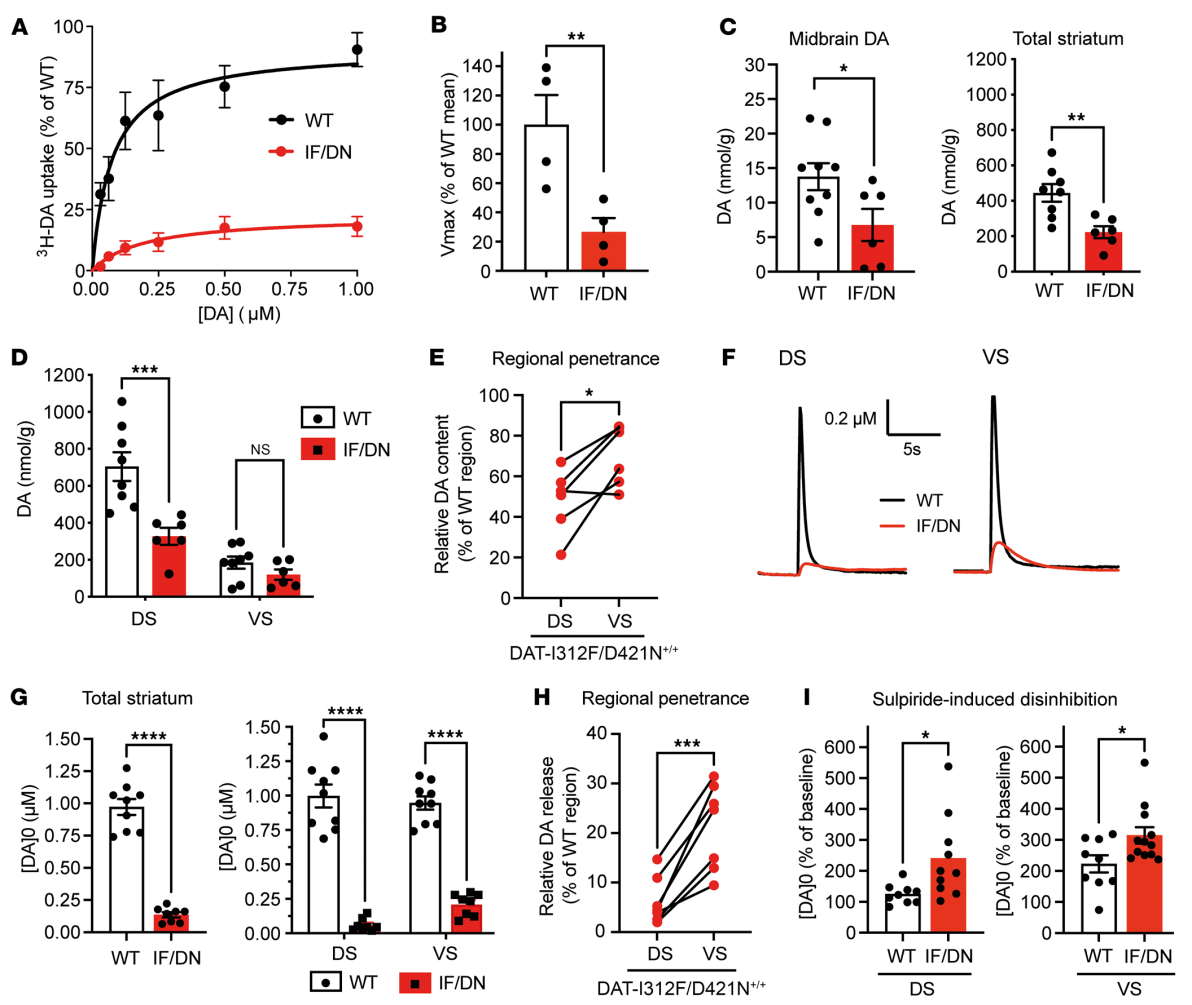
DA dysregulation in DAT-I312F/D421N^+/+^ mice. (**A**) ^3^H-DA saturation uptake on striatal synaptosomes. Data show mean uptake curves from 4 WT and DAT-I312F/D421N^+/+^ (IF/DN) pairs, each normalized to the WT *V_max_*. (**B**) Relative *V_max_* for ^3^H-DA synaptosomal uptake in DAT-I312F/D421N^+/+^ vs. WT mice (*P* < 0.01, paired *t* test, *N* = 4 WT:IF/DN pairs; *K_m_* values: 0.16 ± 0.09 μM for WT vs. 0.25 ± 0.1 μM for I312F/D421N^+/+^, *P* > 0.05, unpaired *t* test). (**C**) HPLC DA tissue content in midbrain (*N* = 9 WT, 6 DAT-I312F/D421N^+/+^ mice, *P* < 0.05, unpaired *t* test) and total striatum (*N* = 8 WT, 6 DAT-I312F/D421N^+/+^ mice, *P* < 0.01, unpaired *t* test). (**D**) Striatal DA content by subregion (DS and VS) in DAT-I312F/D421N^+/+^ mice (*N* = 8 WT, 6 DAT-I312F/D421N^+/+^ mice, 2-way ANOVA with Holm-Šidák correction; DS, *P* < 0.001; VS, *P* > 0.05). (**E**) Regional penetrance of DAT-I312F/D421N^+/+^ genotype on DA content in DS and VS. For each DAT-I312F/D421N^+/+^ mouse, DA content in DS and VS was normalized to mean of WT controls’ corresponding regions (*N* = 6 DAT-I312F/D421N^+/+^ mice, *P* < 0.05, paired *t* test). (**F**) Example FSCV traces of evoked DA release from DS (top) and VS (bottom) in acute striatal slices from WT and DAT-I312F/D421N^+/+^ mice. (**G**) Peak DA concentrations, [DA_0_], averaged across all recording sites per slice (left, WT vs. DAT-I312F/D421N^+/+^, *P* < 0.001, unpaired *t* test) and analyzed by subregion (right, DS vs. VS, *P* < 0.0001, 2-way ANOVA with Holm-Šidák correction). (**H**) Regional penetrance of DAT-I312F/D421N^+/+^ genotype on DA release in DS and VS. Paired DS and VS recordings from DAT-I312F/D421N^+/+^ slices were normalized to the corresponding means from WT (*P* < 0.001, paired *t* test). Data for **G** and **H** are *N* = 9 WT slices (6 mice) and 8 DAT-I312F/D421N^+/+^ slices (5 mice). (**I**) Sulpiride-mediated disinhibition of DA release during train stimulation, expressed as percentage of baseline in DS (left) and VS (right) (*P* < 0.05, unpaired *t* test, *N* = 9 WT slices (6 mice) and 10 (DS) or 12 (VS) DAT-I312F/D421N^+/+^ mouse slices (6 mice). Data are mean ± SEM. *P < 0.05, **P < 0.01, ***P < 0.001, ****P < 0.0001.

Generation of the DAT-I312F/D421N^+/+^ compound heterozygous mice was achieved by crossbreeding of DAT-I312F^+/WT^ heterozygotes with DAT-D421N^+/WT^ heterozygotes (Supplemental Figure 1; supplemental material available online with this article; https://doi.org/10.1172/JCI169297DS1). To directly measure if the mutations have a functional impact on DA uptake in vivo, we first measured ^3^H-DA uptake using striatal synaptosomes. Relative to WT mice, the maximal uptake capacity of DAT-I312F/D421N^+/+^ mice was reduced by approximately 75% (Figure 1, A and B), without significant changes in the apparent DA affinity (*K_m_* WT = 0.16 ± 0.09 μM versus *K_m_* DAT-I312F/D421N^+/+^ = 0.25 ± 0.1 μM, *P* > 0.05, Figure 1A), substantiating the disruptive properties of the disease-associated mutations previously described in vitro.

Next, we measured the total DA tissue content in dorsal striatal (DS), ventral striatal (VS), and midbrain samples. In general, the DA tissue content was diminished by approximately 50% in DAT-I312F/D421N^+/+^ mice in both combined striatum (DS+VS) and midbrain (Figure 1C). Interestingly, however, when analyzing the DS and VS samples separately, we found that the DA tissue content was significantly reduced only in DS but not VS of DAT-I312F/D421N^+/+^ mice (Figure 1D). To directly compare the regional effects of the mutations in the striatum, we normalized the DA content in DS and VS of DAT-I312F/D421N^+/+^ mice to the DA content in corresponding regions of WT control mice. This confirmed that DS was significantly more affected than VS in DAT-I312F/D421N^+/+^ mice (Figure 1E), suggesting that penetrance of the DAT-I312F/D421N^+/+^ genotype is heterogeneous across striatum.

To further assess the impact of the 2 mutations on DA neurotransmission, we used fast-scan cyclic voltammetry (FSCV) to record stimulated DA release and reuptake in striatal slices. We carried out single-pulse stimulations with matched measurements of DA release and reuptake in DS and VS in each slice. Figure 1, F and G, show peak DA concentrations along with representative traces of the recorded DA current in WT mice and DAT-I312F/D421N^+/+^ mice. Across the striatum, evoked DA release was reduced by more than 80% in DAT-I312F/D421N^+/+^ mice compared with WT mice (Figure 1G). Analysis of DA kinetics showed clear increases in both time to peak (TTP; ~2.5 fold) and halftime of clearance (*t_1/2_*; ~5 fold) in DAT-I312F/D421N^+/+^ mice, consistent with a profound impairment in DAT function (Supplemental Table 1). Interestingly, when we compared the functional deficits from matched recordings in DS and VS from each DAT-I312F/D421N^+/+^ striatal slice to the corresponding regions in WT mice, DA release was significantly more compromised in DS than in VS (6.0% ± 2% of WT in DS versus 22% ± 3% of WT in VS, *P* < 0.001; Figure 1, G and H). Likewise, both TTP and the halftime of DA clearance were relatively longer in DS than in VS of DAT-I312F/D421N^+/+^ mice (Supplemental Table 1), further supporting a region-specific penetrance of the disease-associated DAT variants. Noting that the reduction in evoked DA release was more substantial than the decrease in total DA tissue content, we hypothesized that the disease-associated DAT variants would not only alter the distribution of DA between intracellular and extracellular compartments but also promote D2 autoreceptor-mediated inhibition of DA release via elevated extracellular DA tone (25). To address this, we conducted paired recordings of DA release during train stimulations in the absence and presence of the D2 receptor (DR2) antagonist, sulpiride, to unmask D2-mediated autoinhibition of DA release (26, 27) (Figure 1I). Intriguingly, sulpiride produced larger disinhibition of DA release in striatal slices from DAT-I312F/D421N^+/+^ mice compared with WT in both DS and VS (Figure 1I), supporting the hypothesis that increased tonic activation of DR2 in DAT-I312F/D421N^+/+^ mice contributes to the observed deficiencies in stimulated DA release.

### DAT-I312F/D421N^+/+^ mice show synaptopathy with terminal loss and DA receptor downregulation.

**Figure 2:**
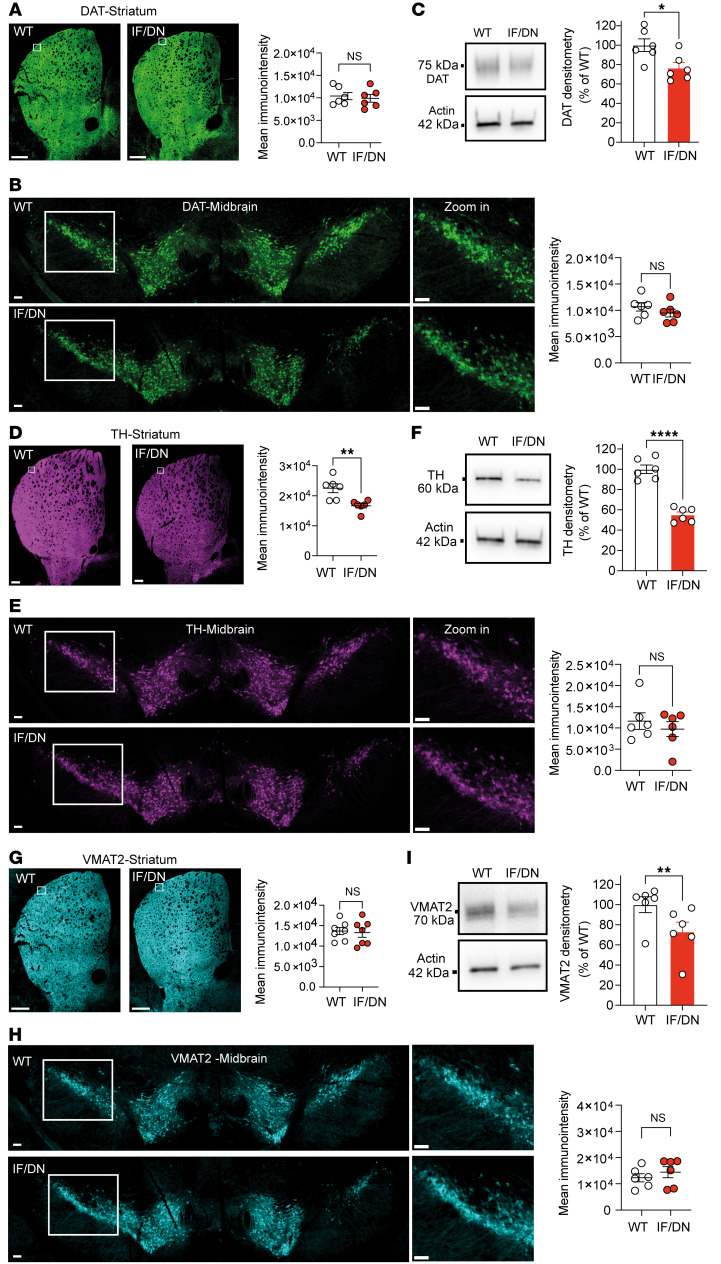
Expression of DAT, TH, and VMAT2 in midbrain and striatum of DAT-I312F/D421N^+/+^ mice. (**A** and **B**) Immunohistochemical visualization by wide-field imaging of DAT in coronal striatal (**A**) and midbrain sections (**B**) of WT and DAT-I312F/D421N^+/+^ (IF/DN) mice with quantification of mean fluorescence intensities (*P* > 0.05, ratio-paired *t* test. *N* = 6 WT:IF/DN pairs. Each WT:IF/DN pair was independently immunolabeled and imaged in parallel). (**C**) Quantification of striatal DAT protein levels by Western blotting of crude synaptosomal preparations from DAT-I312F/D421N^+/+^ mice relative to WT controls (*P* < 0.05, ratio-paired *t* test. *N* = 6 WT:IF/DN pairs. Each pair was processed and analyzed in parallel). (**D** and **E**) Immunohistochemical visualization by wide-field imaging of TH in coronal striatal (**D**) and midbrain sections (**E**) of WT and DAT-I312F/D421N^+/+^ mice with quantification of mean fluorescence intensities (*P* < 0.01 for striatum and *P* > 0.05 for midbrain, *N* = 6 WT:IF/DN pairs, ratio-paired *t* tests). (**F**) Quantification of striatal TH protein levels by Western blotting of crude synaptosomal preparations from DAT-I312F/D421N^+/+^ mice relative to WT controls (*P* < 0.0001, ratio-paired *t* test. *N* = 6 WT:IF/DN pairs). (**G** and **H**) Immunohistochemical visualization by wide-field imaging of VMAT2 in coronal striatal (**G**) and midbrain sections (**H**) of WT and DAT-I312F/D421N^+/+^ mice with quantification of mean fluorescence intensities (*P* > 0.05, ratio-paired *t* tests. *N* = 7 WT:IF/DN pairs for striatum. *P* > 0.05. *N* = 6 WT:IF/DN pairs for midbrain). (**I**) Quantification of striatal VMAT2 protein levels by Western blotting of crude synaptosomal preparations from DAT-I312F/D421N^+/+^ mice relative to WT controls (*P* < 0.01, ratio-paired *t* test. *N* = 6 WT:IF/DN pairs). Data are mean ± SEM. Scale bars: striatum overview: 100 μm; striatum zoom-in and midbrain images: 10 μm. *P < 0.05, **P < 0.01, ****P < 0.0001.

Impaired DA uptake in DAT-I312F/D421N^+/+^ mice could reflect either reduced striatal protein levels or direct impairment of transport activity. To assess the expression and striatal targeting of the disease-associated DAT variants, we visualized DAT in striatal and midbrain slices by immunohistochemistry. In striatal slices, we observed a clear DAT immunosignal in both WT and DAT-I312F/D421N^+/+^ slices without a significant difference in mean labeling intensity across striatum (Figure 2A). Likewise, staining of midbrain slices showed comparable DAT immunosignal from DAergic neurons in both genotypes (Figure 2B). These findings indicate, in agreement with previous in vitro studies (23), that the DAT-I312F and DAT-D421N mutations do not cause an overall folding deficiency with ER retention and loss of striatal targeting. As a further investigation of striatal DAT protein levels, we performed Western blotting on striatal synaptosomes. We observed approximately 23% decrease in total DAT protein levels in DAT-I312F/D421N^+/+^ mice (Figure 2C), which was not apparent from the immunohistochemistry images. This finding was further validated when we performed surface biotinylation experiments on slices from DS, which showed a reduction in total DAT levels in DAT-I312F/D421N^+/+^ mice, whereas the surface/total ratio was unchanged (Supplemental Figure 2). Interestingly, similar experiments on slices from VS did not show significant changes in total or surface DAT protein levels, further indicating that DA projections to DS appear particularly sensitive to the DAT-I312F/D421N mutations (Supplemental Figure 2). We also assessed the expression of tyrosine hydroxylase (TH), the rate-limiting enzyme in DA synthesis, and the vesicular monoamine transporter 2 (VMAT2), which mediates DA sequestration into synaptic vesicles. Here, we observed a reduction in the mean TH immunosignal in striatal slices, but no change in midbrain slices, from DAT-I312F/D421N^+/+^ mice compared with WT littermates (Figure 2, D and E). This finding was further confirmed by Western blotting of striatal synaptosomes and total protein lysates, which showed an approximately 45% decrease in striatal TH expression in DAT-I312F/D421N^+/+^ mice (Figure 2F and Supplemental Figure 2). As observed for DAT, widefield fluorescence imaging of VMAT2 in striatal and midbrain slices showed no apparent genotype differences (Figure 2, G and H), while Western blotting revealed a significant reduction in striatal VMAT2 protein levels of approximately 30% in I312F/D421N^+/+^ mice (Figure 2I). Of notice, we did not observe genotype differences in DAT, TH, and VMAT2 mRNA levels in midbrain lysates, indicating that reduced protein expression of DAT, TH, and VMAT2 in striatum was not driven by transcriptional changes (Supplemental Figure 2).

**Figure 3:**
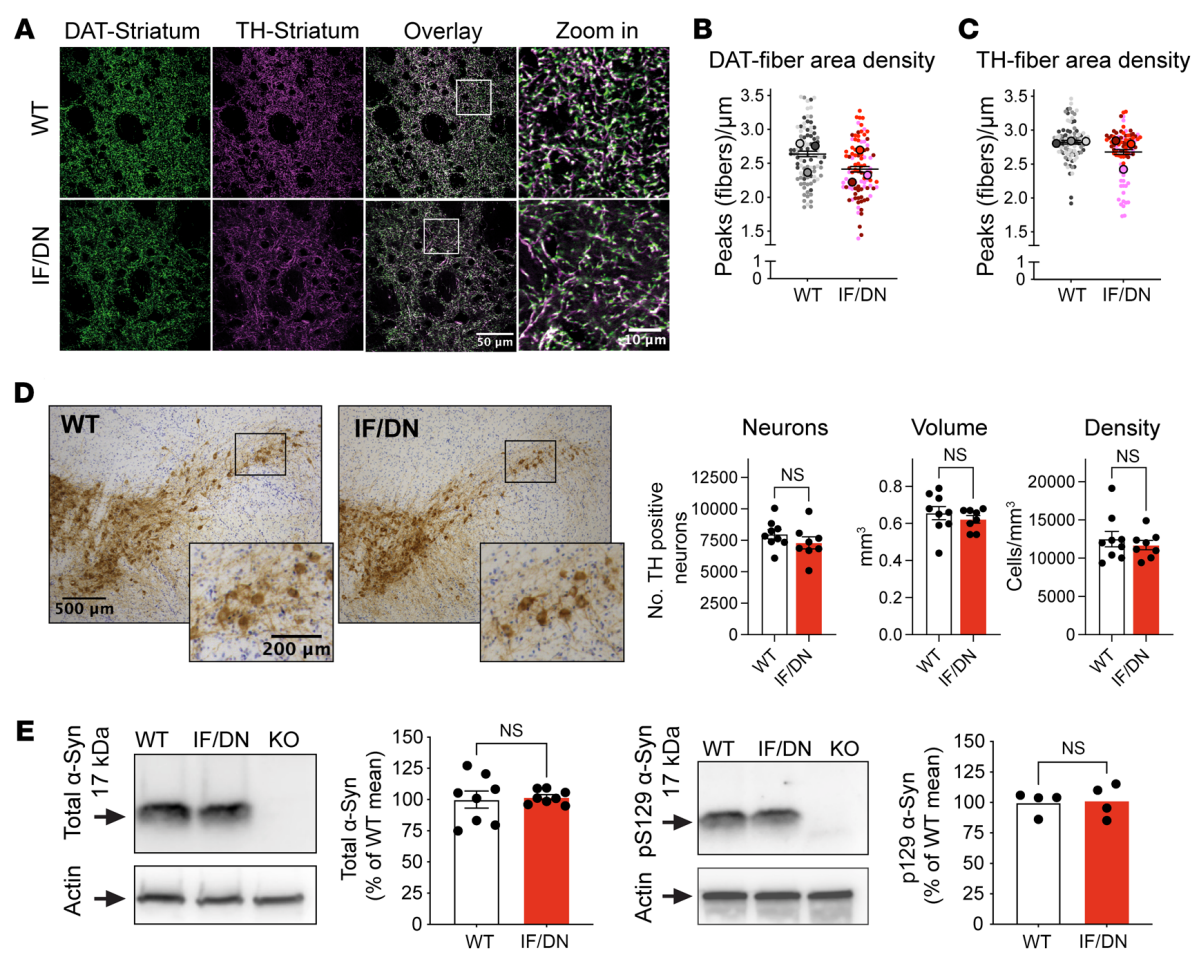
Reduced DAergic terminal density in DAT-I312F/D421N^+/+^ mice. (**A**) Representative confocal images of striatal DA projections immunolabeled for DAT and TH. (**B** and **C**) Quantification of area density of (**B**) DAT- and (**C**) TH-labeled fibers in DAT-I312F/D421N^+/+^ (IF/DN) mice compared with WT shown as SuperPlots (small points represent images; large points represent mouse means). Images were systematically sampled from striatal slices from 3 independent WT:IF/DN pairs, stained and imaged in parallel, and analyzed blinded to genotype. Image-level statistic shows significant reductions in both DAT and TH area density (*P* < 0.001 for DAT, *N* = 96 WT and 96 IF/DN; *P* < 0.01 for TH, unpaired *t* test, *N* = 95 WT and 90 IF/DN images). Mouse-level statistics: DAT *P* = 0.165; TH *P* = 0.37, *N* = 3, paired *t* test). (**D**) Stereological counting of TH-positive cells. Left, representative visualization of TH-positive cells by DAB staining. Right, quantification of neurons, volume, and density in the substantia nigra of WT and DAT-I312F/D421N^+/+^ mice (*P* > 0.05, unpaired *t* test. *N* = 9 WT and 8 IF/DN mice. See also Supplemental Tables 2 and 3). (**E**) Assessment of α-synuclein (α-Syn) levels in midbrain lysates from WT and DAT-I312F/D421N^+/+^ mice by Western blotting. Left, representative gel and quantification of total α-Syn soluble monomers (*N* = 8 WT:IF/DN pairs). Right, representative gel and quantification of phosphorylated α-Syn soluble monomers (pS129, *N* = 4 WT:IF/DN pairs) (*P* > 0.05, ratio-paired *t* test). Data are mean ± SEM.

The parallel reduction in striatal protein levels of DAT, VMAT2, and TH in DAT-I312F/D421N^+/+^ mice found by Western blot analysis of whole striatal lysates led us to investigate if the DAT-I312F/D421N^+/+^ mice display structural changes in DA terminals, which may not be resolved in widefield fluorescence images. We therefore systematically acquired confocal images from striatal slices labeled for DAT and TH to visualize the DAergic arbor in more detail. As expected, the 2 DAergic markers showed almost complete overlap in both WT and DAT-I312F/D421N^+/+^ mice (Figure 3A). The higher resolution confocal images revealed an apparent reduction in the area density of DAergic terminals in images from DAT-I312F/D421N^+/+^ mice compared with WT mice. To directly quantify the area density of DAergic terminals, we applied a line-scan intensity analysis to count the terminals intersecting with a line grid in each image (28) (see Supplemental Methods). In agreement with the visual impression, image-level quantification showed reduced area density of both DAT- and TH-positive terminals in the DAT-I312F/D421N^+/+^ mice compared with WT mice (Figure 3, B and C). These data suggest that the parallel reduction in striatal levels of DAergic markers that we observed by Western blot analysis may be explained, at least in part, by axonal loss in DAT-I312F/D421N^+/+^ mice and, thus, that genetic injury to DAT can compromise the microscale integrity of DAergic axon terminals. To determine whether the observed deficit in fiber density arises during development, we examined the striatal terminal density in neonatal pups. Interestingly, across the sampled images, the area density of both TH- and DAT-positive striatal fibers was reduced in DAT-I312F/D421N^+/+^ pups (Supplemental Figure 3). As we noticed that these reductions were proportionally smaller than those observed in adult mutant mice, these data suggest that the reduced density of DAergic terminals reflects, at least in part, a developmental impairment in terminal formation or maintenance.

Despite the deficit in axon fiber area density in striatal images, stereological counting of DAergic neurons in substantia nigra (SN) of the adult mice did not show any genotype differences in cell count, volume, or density (Figure 3D and Supplemental Tables 2 and 3).We also found comparable levels of both unphosphorylated and phosphorylated (p-S129) soluble α-synuclein monomers in midbrain lysates, indicating that the loss of striatal DA terminals in DAT-I312F/D421N^+/+^ mice is not associated with α-synuclein pathology or cell body degeneration (Figure 3E).

**Figure 4:**
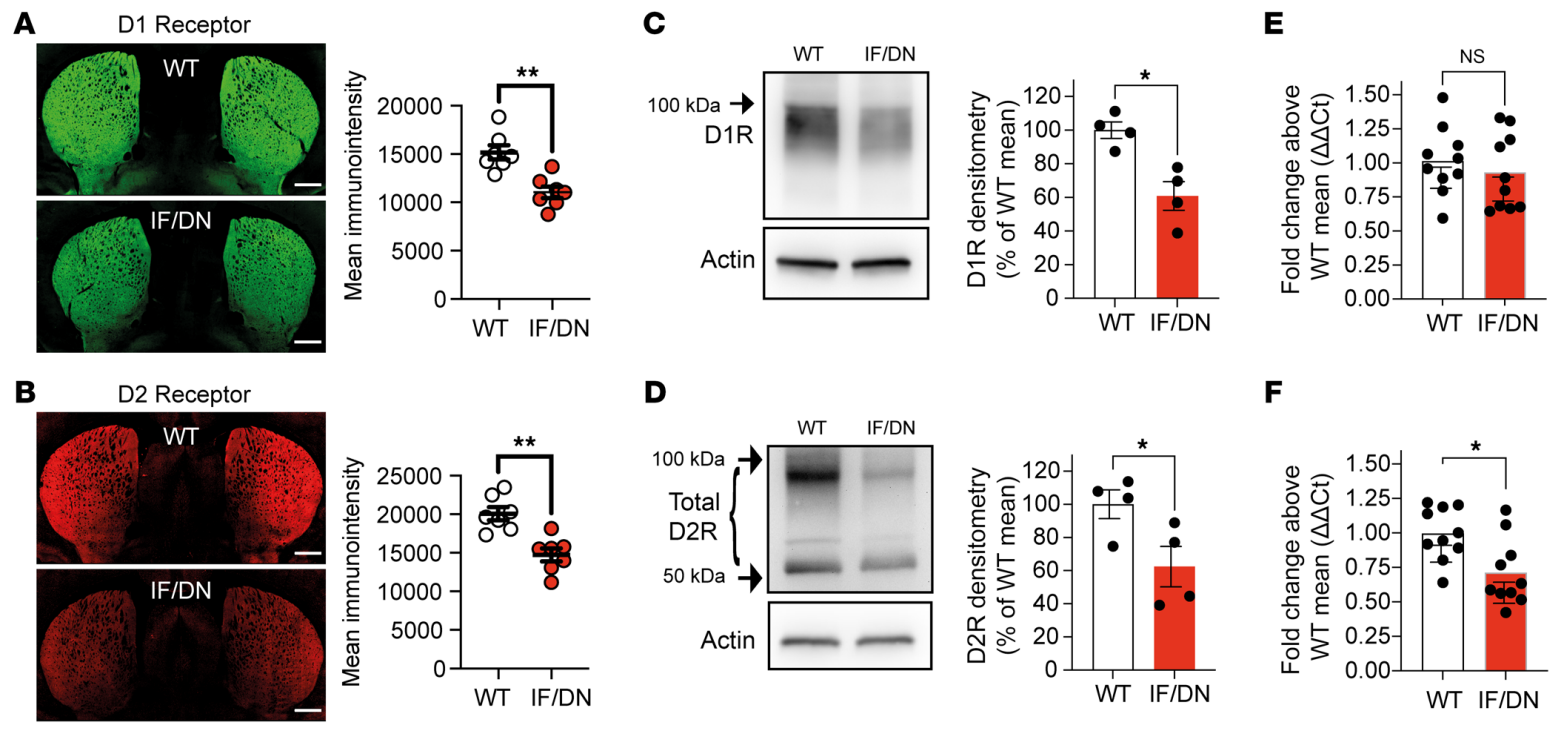
Synaptic adaptations in DAT-I312F/D421N^+/+^ mice. (**A** and **B**) Immunohistochemical visualization (representative images) of DA D1R (**A**) and D2R (**B**) by wide-field imaging on coronal striatal brain slices from pairs of WT and DAT-I312F/D421N^+/+^ mice (see refs. 29 and 30 for knockout validation of antibodies) with quantification of mean fluorescence intensity (*P* < 0.01, ratio-paired *t* test. *N* = 7 WT:IF/DN pairs that were independently labeled and imaged in parallel. (**C** and **D**) D1R and D2R Western blot analysis on DS homogenates from pairs of WT and DAT-I312F/D421N^+/+^ mice (left, representative blots; right, quantification; *P* < 0.05, ratio-paired *t* test. *N* = 4 WT:IF/DN pairs, each processed and analyzed in parallel). (**E** and **F**) D1R and D2R mRNA levels in DS lysates by quantitative real-time PCR shown as fold-change above the WT mean (2^-ΔΔCt^, *P* > 0.05 for D1R, *P* < 0.05 for D2R, *N* = 10 mice per genotype, multiple unpaired *t* test with Holm-Šidák correction). Data are mean ± SEM. Scale bar: 300 μm. *P < 0.05, **P < 0.01.

Changes in DA release and in DAergic axon terminal density in the striatum could lead to additional adaptations in the expression of DA D1 receptor (DR1) and DR2 in the striatum. We thus examined this using knockout-validated antibodies and qPCR (29, 30). Immunostaining of striatal slices showed decreased immunolabeling intensity of both DR1 and DR2 in DAT-I312F/D421N^+/+^ mice compared with WT mice (Figure 4, A and B). This reduction in striatal DR1 and DR2 protein levels was also observed upon Western blotting of lysates from DS (Figure 4, C and D). To determine if the changes in DR1 and DR2 protein levels involved transcriptional changes, we quantified mRNA levels in DS lysates by qPCR. Interestingly, we found a significant reduction in DR2 mRNA levels in DAT-I312F/D421N^+/+^ mice, whereas DR1 mRNA levels were comparable to WT mice, indicating that the reduction in DR2, but not DR1, protein levels involves transcriptional changes (Figure 4, E and F). As we did not find changes in DR2 mRNA levels in midbrain lysates, these changes in DS DR2 mRNA levels (Supplemental Figure 2A) presumably reflect postsynaptic adaptations.

Taken together, our findings demonstrate that the genetic insult to DAT function causes a DA dysfunction that extends beyond DA reuptake and release to also affect DA synthesis, postsynaptic receptor levels, and the microscale integrity of DAergic axonal arborization.

### DAT-I312F/D421N^+/+^ mice display persistent hyperactivity and increased explorative behavior.

**Figure 5:**
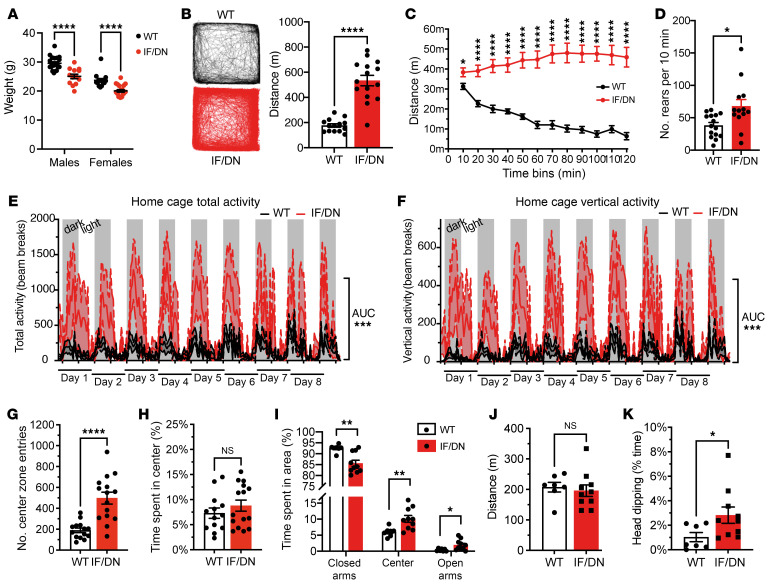
DAT-I312F/D421N^+/+^ mice display persistent hyperactivity and increased exploratory behavior. (**A**) Body weight for male and female WT and DAT-I312F/D421N^+/+^ (IF/DN) mice (*P* < 0.0001, 2-way ANOVA with Holm-Šidák’s correction, *N*_males_ = 20 WT, 13 IF/DN. *N*_females_ = 14 WT, 20 IF/DN). (**B**) Locomotor activity during a 2-hour open-field test (OFT); left, mouse trajectory plots; right, total distance moved (*P* < 0.0001, Welch’s *t* test, *N* = 15 IF/DN and 14 WT). (**C**) Locomotor activity during the 2-hour OFT across 10-minute intervals (*P* < 0.001, multiple Welch’s *t* test with Holm-Šidák’s correction for multiple comparisons, *N* = 15 IF/DN and 14 WT). (**D**) Rearing during 10-minute cylinder test (*P* < 0.05, Welch’s *t* tests, *N* = 15 WT and 13 IF/DN mice). (**E** and **F**) Home cage activity recordings of total (**E**) and vertical (**F**) locomotor activity (dark phases in gray). Total activity in the home cage environment, quantified as AUC (*P* < 0.001, Welch’s *t* test, **E**: *N* = 7 WT and 7 IF/DN; **F**: *N* = 7 WT and 6 IF/DN mice). (**G**) Center zone entries during the 2-hour OFT (*P* < 0.0001, Welch’s *t* test, *N* = 14 WT and 15 IF/DN). (**H**) Time spent in center zone (%) during the 2-hour OFT (*P* > 0.05, Mann-Whitney *U* test, *N* = 13 WT and 15 IF/DN). (**I**) Elevated plus maze (EPM): % time spent in closed arms, center zone, and open arms for DAT-I312F/D421N^+/+^ versus WT mice (multiple Welch’s *t* test with Holm-Šidák’s correction, *N* = 7 WT and 10 IF/DN mice). (**J**) Distance traveled in the EPM (*P* > 0.05, Welch *t* test, *N* = 7 WT and 10 IF/DN mice). (**K**) Head-dipping in the EPM (*P* < 0.05, Welch’s *t* test, *N* = 7 WT and 10 IF/DN mice). All data are shown as mean ± SEM. *P < 0.05, **P < 0.01, ***P < 0.001, ****P < 0.0001.

We next characterized the overall behavioral phenotype of the DAT-I312F/D421N^+/+^ mice. These analyses were performed on adult male and female mice during a 5-week period starting at age 15 ± 2 weeks. Of note, both male and female DAT-I312F/D421N^+/+^ mice were, by visual inspection, smaller than WT littermates. Compared with WT mice, the body weight was on average 4.6 g lower in DAT-I312F/D421N^+/+^ males (15.6%) and 3.6 g lower (15.2%) in females (Figure 5A).

Disturbances in DAergic neurotransmission have been widely implicated in ADHD, and the patient modeled by the DAT-I312F/D421N^+/+^ mice was diagnosed with ADHD in addition to atypical parkinsonism (23). A core symptom of ADHD is hyperactivity, and we therefore compared spontaneous locomotor activity of DAT-I312F/D421N^+/+^ mice with WT littermates in a 2-hour, novel open-field test. On average, the DAT-I312F/D421N^+/+^ mice traveled more than 3 times the distance of their WT littermates (Figure 5B). This hyperlocomotive behavior of DAT-I312F/D421N^+/+^ mice was evident throughout the entire 2-hour test period, but became more pronounced over time, as the WT mice habituated to the novel environment during 2 hours of recording while the DAT-I312F/D421N^+/+^ mice showed persistently high locomotor activity (Figure 5C and Supplemental Videos 1–4). We compared the rearing behavior of WT and DAT-I312F/D421N^+/+^ mice in a novel environment by counting the rears after placing the mice in a glass cylinder for 10 minutes. The DAT-I312F/D421N^+/+^ mice displayed nearly twice as many rears as the WT mice (Figure 5D), collectively demonstrating that the DAT-I312F/D421N^+/+^ mice have both increased horizontal and vertical activity in novel environments.

To assess if the observed hyperactivity was novelty dependent, we performed extended activity recordings of DAT-I312F/D421N^+/+^ and WT mice in activity cages. During 8.5 days of monitoring, the DAT-I312F/D421N^+/+^ mice displayed persistently higher activity than WT mice both in total activity (Figure 5E) and in vertical activity (Figure 5F), showing that the hyperlocomotive phenotype of DAT-I312F/D421N^+/+^ mice is present even in the absence of novelty.

We next examined the effect of genotype on explorative versus anxiety-related phenotypes by analyzing the number of center zone entries and time spent in the center zone in the open-field test. The DAT-I312F/D421N^+/+^ mice showed a 2.6-fold increase in the number of center zone entries (Figure 5G), but no difference in percentage time spent in center zone (Figure 5H), indicating no anxiety-related center zone aversion. We further tested the mice for their propensity to explore versus their tendency to seek protected areas in an elevated plus maze (EPM) paradigm. We found that the DAT-I312F/D421N^+/+^ mice spent more time in the center zone and open arms than WT mice (Figure 5I). Of notice, this increase was not caused by an overall increase in locomotion, as the distance traveled by DAT-I312F/D421N^+/+^ mice during the test was not increased compared with WT mice (Figure 5J). Consistent with an increased explorative drive, the DAT-I312F/D421N^+/+^ mice also spent almost 3-fold more time engaged in head dipping over the sides of the open arms (Figure 5K), which is an additional behavioral correlate of explorative activity (31).

In summary, the DA imbalance imposed by the disease-associated mutations in DAT drives an ADHD-relevant phenotype characterized by hyperactivity and an enhanced exploratory drive.

### DAT-I312F/D421N^+/+^ mice show increased hind limb clasping and kyphosis.

**Figure 6:**
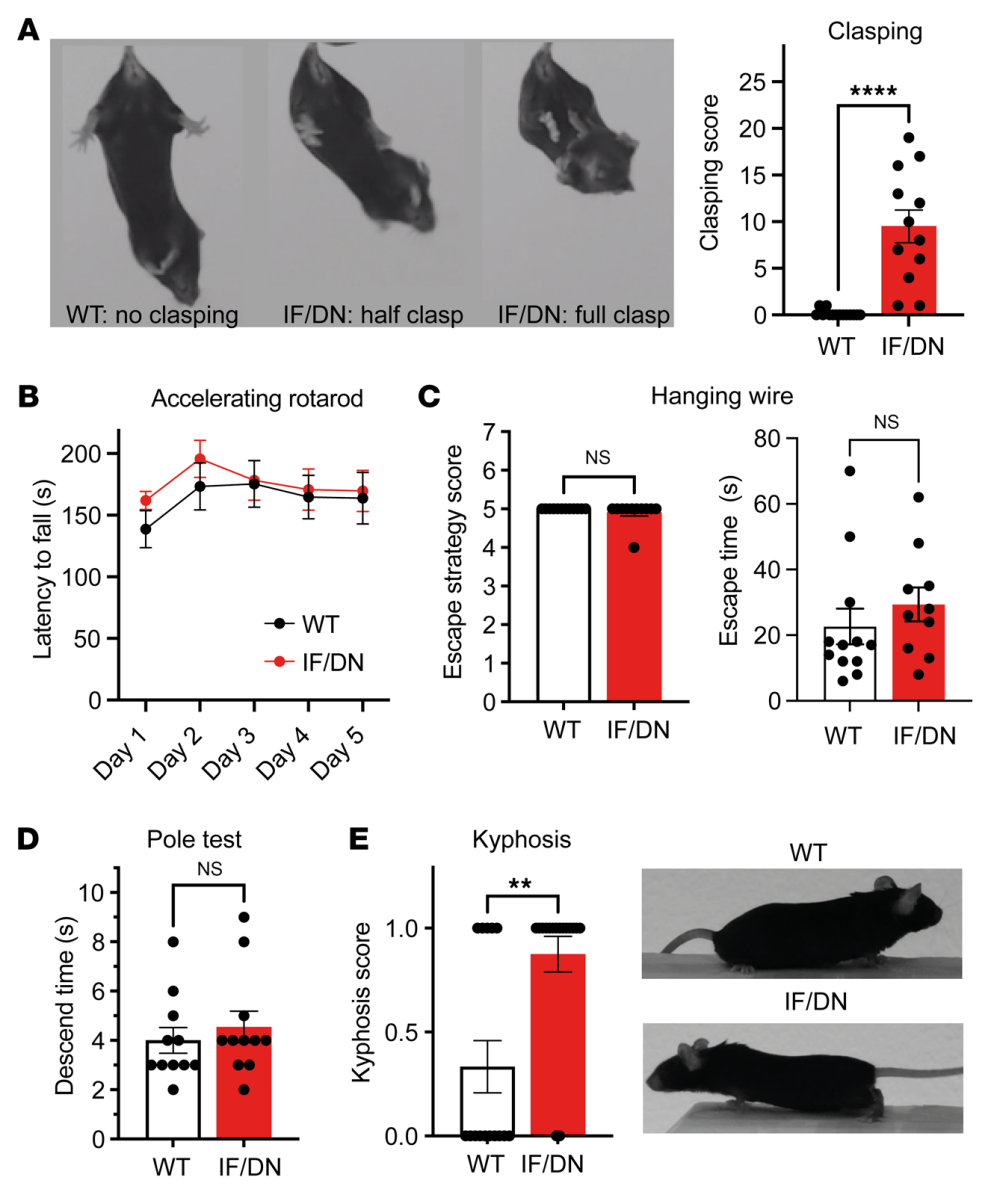
DAT-I312F/D421N^+/+^ mice exhibit clasping behavior and kyphosis. (**A**) Representative images of hind limb clasping phenotypes in DAT-I312F/D421N^+/+^ mice during tail suspension with quantification of clasping score (see Methods; *P* < 0.0001, Mann-Whitney *U* test. *N* = 14 WT and 12 IF/DN mice). (**B**) Accelerating rotarod test (*P* > 0.05 for all test days by 2-way ANOVA with Holm-Šidák multiple comparisons test. *N* = 13 WT and 12 IF/DN mice). (**C**) Hanging wire test with escape strategy scores, left (*P* > 0.05, Mann-Whitney *U* test. *N* = 12 WT and 11 IF/DN mice); and escape time to platform, right (*P* > 0.05, Mann-Whitney *U* test. *N* = 12 WT and 10 IF/DN mice). (**D**) Pole test descent time (*P* > 0.05, Mann-Whitney *U* test. *N* = 11 WT and 11 IF/DN mice). (**E**) DAT-I312F/D421N^+/+^ received higher scores in visual inspection for dorsal kyphosis than WT mice (*P* < 0.01, Mann-Whitney *U* test. *N* = 22 WT and 22 IF/DN mice). All data are shown as mean ± SEM. **P < 0.01, ****P < 0.0001.

Transgenic mouse models expressing genetic variants associated with hereditable forms of PD display notoriously few, if any, motor deficits (32–35). To assess the motor function of DAT-I312F/D421N^+/+^ mice, we performed a battery of motor tests comprising rotarod, pole test, hanging wire test, and hind limb clasping during tail suspension. Most prominently, we observed a clear motor abnormality in the DAT-I312F/D421N^+/+^ mice when assessing hind limb clasping. While DAT-WT mice showed practically no clasping behavior when lifted by their tail for 30 seconds, the DAT-I312F/D421N^+/+^ mice displayed clear, but varying, degrees of clasping, seen as partial or full retraction of hind limbs toward the midline in a dystonic fashion (Figure 6A and Supplemental Videos 5 and 6). This clasping phenotype of DAT-I312F/D421N^+/+^ mice was not accompanied by reduced motor performance on an accelerating rotarod, in the pole test, or in the hanging wire test (Figure 6, B–D), suggesting that DAT-I312F/D421N^+/+^ mice can acquire and execute motor tasks to a similar level of performance as WT mice. Nonetheless, in addition to receiving higher clasping scores, the DAT-I312F/D421N^+/+^ mice also received higher scores when visually inspected for dorsal kyphosis (Figure 6E), a phenotype that has been linked to premature aging and movement disorders (36–38).

### Hyperactivity in DAT-I312F/D421N^+/+^ mice is ameliorated by amphetamine and orphenadrine.

**Figure 7:**
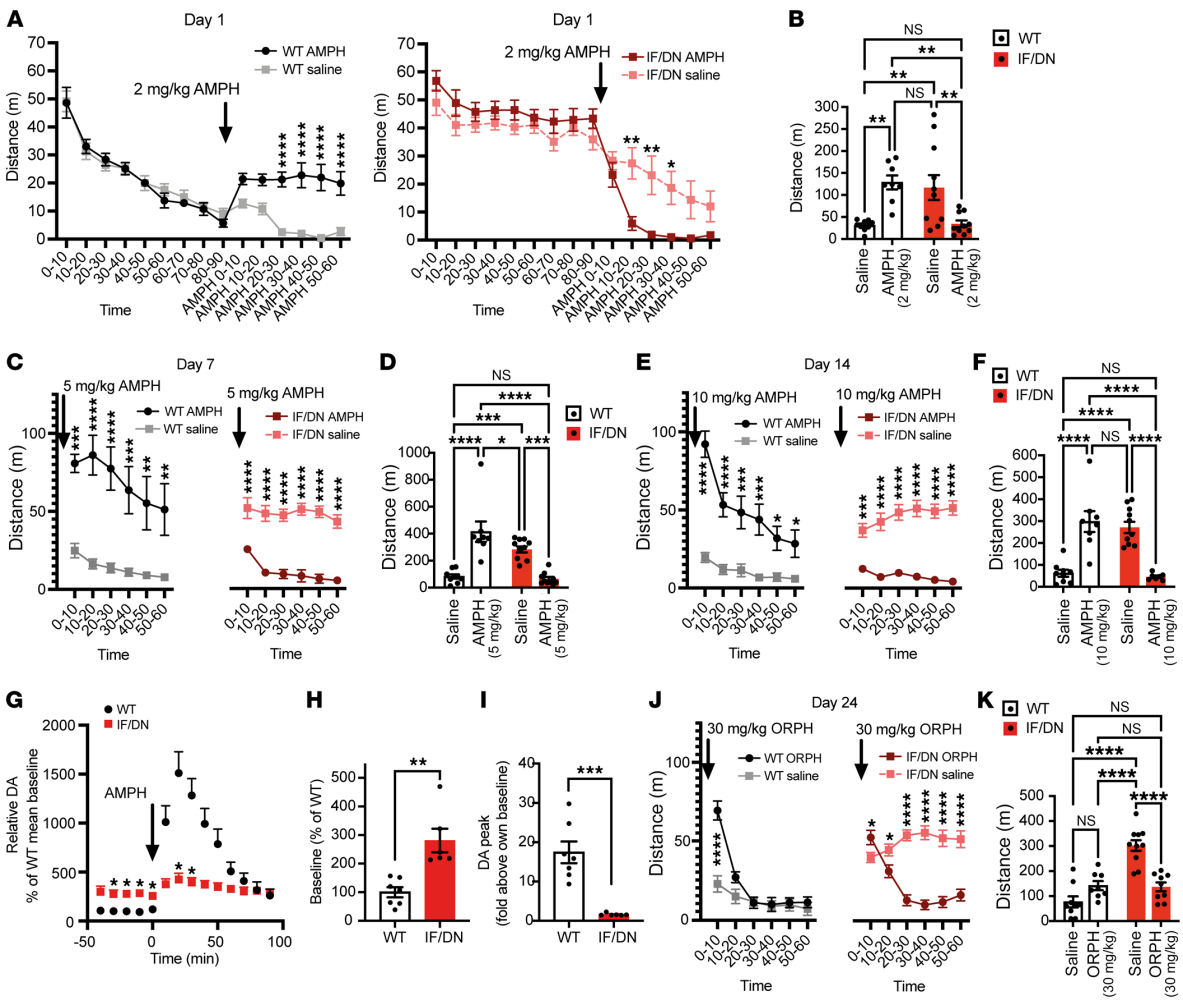
AMPH and orphenadrine alleviate hyperactivity in DAT-I312F/D421N^+/+^ mice. (**A**) Open-field locomotion before and after AMPH (2 mg/kg, i.p.) in WT and DAT-I312F/D421N^+/+^ (IF/DN) mice (RM 2-way ANOVA with Holm-Šidák correction). (**B**) Total distance traveled after saline or AMPH (2 mg/kg) injections (2-way ANOVA with Holm-Šidák correction, *N* = 9 WT_saline_, 8 WT_AMPH_, 10 IF/DN_saline_, and 10 IF/DN_AMPH_). (**C**–**F**) Reexposure to saline or 5 mg/kg AMPH (**C** and **D**) and saline or 10 mg/kg AMPH (**E** and **F**) after 7 days’ washout. Locomotion is shown in 10-minute bins (**C** and **E**; RM 2-way ANOVA with Holm-Šidák correction) and as total distance over 1 hour after injections (**D** and **F**, 2-way ANOVA with Holm-Šidák correction, *N* = 9 WT_saline_, 10 IF/DN_saline_, 8 WT_5 mg/kg_, 10 IF/DN_5 mg/kg_, 8 WT_10 mg/kg_, 7 IF/DN_10 mg/kg_). (**G**) Slow-flow microdialysis of extracellular striatal DA in anesthetized WT and DAT-I312F/D421N^+/+^ mice before and after AMPH (2 mg/kg, i.p.). Data are expressed as percentage of WT mean baseline (mixed-effect model with Holm-Šidák correction, *N* = 7 WT and 6 IF/DN mice). (**H**) Relative baseline DA levels in percentage of WT mean (*P* < 0.01, unpaired *t* test, *N* = 7 WT and 6 IF/DN mice). (**I**) Peak AMPH-induced DA levels in WT and DAT-I312F/D421N^+/+^ mice (*P* < 0.001, unpaired *t* test, *N* = 7 WT and 6 IF/DN mice). (**J**) Open-field locomotion in 10-minute time bins after ORPH (30 mg/kg, i.p.) administration to WT and DAT-I312F/D421N^+/+^ mice (RM 2-way ANOVA with Holm-Šidák correction). (**K**) Total distance traveled after saline or ORPH (30 mg/kg) (2-way ANOVA with Holm-Šidák correction). *N* for **J** and **K** = 9 WT_saline_, 8 WT_ORPH_, 10 IF/DN_saline_, 9 IF/DN_ORPH_. All data are shown as mean ± SEM. *P < 0.05, **P < 0.01, ***P < 0.001, ****P < 0.0001.

Hyperactivity is a core symptom of ADHD and recapitulated in the DAT-I312F/D421N^+/+^ mouse. Since amphetamine (AMPH) is frequently used to treat ADHD in humans, we next evaluated the effect of AMPH on spontaneous locomotion in an open-field arena. Following a 1-hour habituation period, DAT-I312F/D421N^+/+^ mice and WT littermates were first treated with either a single dose of AMPH (2 mg/kg) or a saline injection, after which activity was recorded for an additional 1 hour. For AMPH-treated WT mice, we observed, as expected, a significant AMPH-induced increase in the distance traveled compared with saline controls (Figure 7, A and B). In striking contrast, the hyperactive behavior of DAT-I312F/D421N^+/+^ mice was ameliorated by AMPH treatment, showing a reduction in traveled distance (Figure 7, A and B).

We previously showed that both the DAT-D421N and the DAT-I312F mutations alter DAT’s substrate and inhibitor binding properties (23, 39). To exclude the possibility that higher doses of AMPH were required to elicit AMPH-induced hyperlocomotion in DAT-I312F/D421N^+/+^ mice, we reexposed the mice to 5 mg/kg AMPH after a 1-week washout period and again to 10 mg/kg AMPH after a second 1-week washout period. Whereas WT littermates showed even more robust increases in locomotion in response to 5 mg/kg and 10 mg/kg AMPH (Figure 7, C–F), the DAT-I312F/D421N^+/+^ mice still demonstrated a pronounced decrease in activity at both 5 mg/kg and 10 mg/kg AMPH (Figure 7, C–F), further consolidating the attenuating effect of AMPH on the hyperactive DAT-I312F/D421N^+/+^ phenotype.

To gain further insights into the effect of AMPH on DAT-I312F/D421N^+/+^ mice, we carried out slow-flow microdialysis experiments on anesthetized mice to compare baseline DA levels and AMPH-induced DA release in the DS. Baseline DA levels were measured over a 40-minute time window before i.p. administration of 2 mg/kg AMPH (Figure 7G). Importantly, baseline DA measurements revealed that extracellular DA levels in DAT-I312F/D421N^+/+^ mice were 2.8- ± 0.4-fold higher than in WT mice, demonstrating that the DAT-I312F/D421N^+/+^ genotype causes a chronic increase in extracellular DAergic tone (Figure 7H). Administration of AMPH (2 mg/kg) elicited, as expected, a marked increase in extracellular DA in WT mice (Figure 7, G and I). More surprisingly, AMPH also evoked an increase in extracellular DA levels in DAT-I312F/D421N^+/+^ mice (Figure 7, G and I). However, peak DA levels increased 17- ± 2.8-fold above baseline in WT mice whereas only a 1.6- ± 0.13-fold increase above baseline was recorded in DAT-I312F/D421N^+/+^ mice (Figure 7I). Still, these findings suggest that the seemingly calming effect of AMPH on DAT-I312F/D421N^+/+^ mice is not established by a paradoxical decrease in extracellular DA levels leading to a reduced exploratory drive. Rather, the AMPH-treated DAT-I312F/D421N^+/+^ mice are less active despite increased extracellular DA levels.

The index patient with atypical DTDS was treated with the anticholinergic drug, ORPH, to alleviate parkinsonian symptoms. To uncover potential disease-relevant alterations in cholinergic responses (40, 41), the effect of ORPH was evaluated in the open-field test comparing WT and DAT-I312F/D421N^+/+^ mice. Interestingly, analysis of locomotion in 10-minute bins revealed an immediate, but short-lasting (10 minutes), increase in locomotion in ORPH-treated (30 mg/kg) WT mice relative to saline-treated WT mice (Figure 7J). This initial hyperlocomotive response to ORPH was also seen, though less pronounced, in DAT-I312F/D421N^+/+^ mice. However, while ORPH-treated WT mice rapidly resumed activity levels similar to saline-treated mice, DAT-I312F/D421N^+/+^ mice treated with ORPH showed a significant reduction in locomotor activity compared with saline control mice, an effect that persisted throughout the remaining test period (Figure 7J). Thus, during 1-hour recordings, the total distance traveled by ORPH-treated WT mice did not differ significantly from saline-treated mice (Figure 7K), while ORPH alleviated the hyperlocomotive phenotype of DAT-I312F/D421N^+/+^ mice (Figure 7K).

These data further support the predictive validity of the DAT-I312F/D421N^+/+^ mice to identify and validate potential new treatments while pointing to a potentially important cholinergic impairment that likely arises from the primary DA-related dysfunction.

### DAT-I312F/D421N^+/+^ mice show loss of fast DA dynamics and region-specific changes in amphetamine responses.

**Figure 8:**
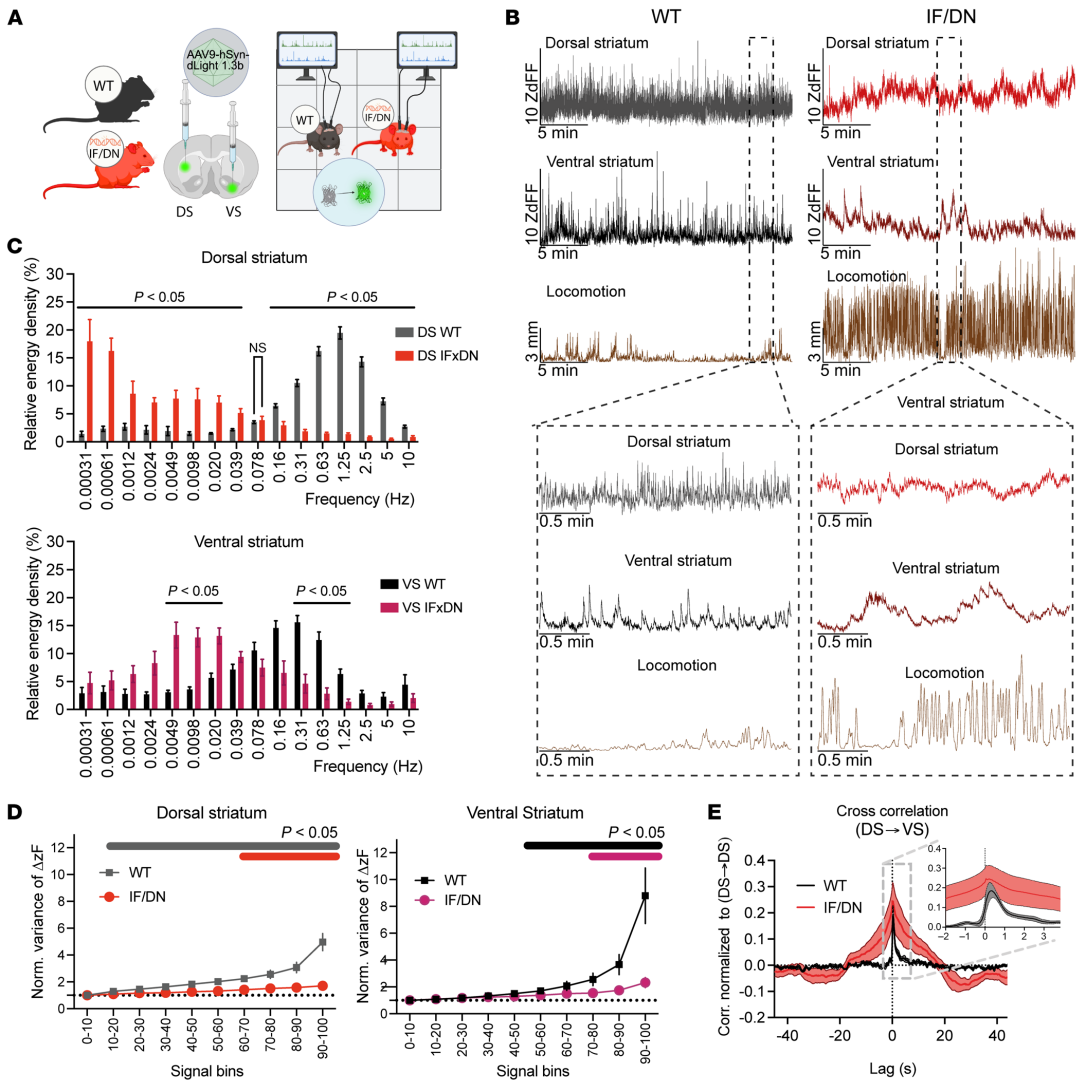
Temporally disrupted and low-complexity striatal DA dynamics in DAT-I312F/D421N^+/+^ mice. (**A**) Fiber photometry with dLight1.3b in dorsal striatum (DS) and ventral striatum (VS) of WT and DAT-I312F/D421N^+/+^ mice during 1-hour, self-paced open-field activity. (**B**) Representative DS and VS DA traces. WT mice show rapid DA fluctuations in DS and transient peaks in VS, whereas DAT-I312F/D421N^+/+^ mice show longer, irregular waves of DA without rapid transients. (**C**) Spectral energy density plots demonstrating decreased high-frequency DA activity and increased low-frequency components in both DS and VS of DAT-I312F/D421N^+/+^ mice (multiple unpaired *t* test with Holm-Šidák correction. *N* = 9 WT and 6 DS or 8 VS IF/DN mice). (**D**) Variance of the first derivative of DA signals across deciles of signal intensity (normalized to the lowest decile) indicates reduced structured, nonrandom DA signals in DAT-I312F/D421N^+/+^ mice (RM2-way ANOVA, followed by within-genotype simple-effect comparisons of deciles relative to baseline, with Benjamini-Hochberg correction. *N* = 9 WT and 6 DS or 8 VS IF/DN mice). (**E**) DS–VS cross-correlation analysis shows a sharp asymmetric peak at approximately 0.35 second lag in WT mice (DS leading), consistent with temporally structured coordination. DAT-I312F/D421N^+/+^ mice exhibit a broader and less defined correlation (*N* = 8 WT and 5 IF/DN), indicating disrupted temporal coupling. Data in **C**–**E** are shown as mean ± SEM.

Recent work has substantiated that temporally precise DA fluctuations are critical for encoding information and shaping striatal output (42–44). We therefore turned to fiber photometry with the genetically encoded DA sensor dLight1.3b (45) to examine how the patient-derived DAT mutations affect the temporal structure and regional coordination of DA signaling and to interrogate the paradoxical response of DAT-I312F/D421N^+/+^ mice to AMPH in further detail. To this end, dLight1.3b was virally expressed in the right DS and left VS of WT and DAT-I312F/D421N^+/+^ mice, and fluorescence signals were recorded more than 3 weeks later during open-field exploration, followed by vehicle or AMPH injections. The DA signal in WT mice during baseline (self-paced exploratory) activity confirmed previous findings, with continuous rapid DA fluctuations in the DS and slower fluctuations in the VS, yet with distinct DA peaks (transients) (42, 46) (Figure 8A). In DAT-I312F/D421N^+/+^ mice, however, the fast DA fluctuations were essentially absent in both DS and VS (Figure 8B). In the DS, the characteristic swift DA peaks were replaced by long, irregular waves overlaid with smaller oscillations, while in the VS, DA signals showed even more prominent, enduring slow waves without the sharp peaks observed WT mice (Figure 8B). Spectral energy density plots across the entire recording period of vehicle-injected mice confirmed, for both DS and VS, a clear reduction in high-frequency DA fluctuations, coupled with the presence of slower, longer lasting DA signals in DAT-I312F/D421N^+/+^ mice (Figure 8C). These results reveal the detrimental effect of the patient-derived mutations on maintaining temporally precise DA signaling.

To evaluate how the changes in DA signals in DAT-I312F/D421N^+/+^ mice affect the potential informational content of the DA fluctuations, we examined the degree to which the signals differed from random variations arising from a homogeneous Poisson process. We previously showed that the DA signals in both DS and VS of WT mice are not derived from random variations but are heterogeneous, supporting that the signals may encode behaviorally relevant information (42). We demonstrated this by computing the variance of the first derivative of the DA signal intensity, as this should be constant across signal intensities (binned into deciles) in a homogeneous process (42). In agreement with these previous data, and consistent with a heterogeneous release pattern, the variance of the first derivative in WT mice was significantly larger for all deciles compared with the bottom 10th percentile in DS and for the upper half of the signal in VS (Figure 8D). In contrast, DA signals in DAT-I312F/D421N^+/+^ mice were homogeneous across a wider range of signal intensities than in WT mice and showed reduced variability even in the bins with significant heterogeneous variation. This supports that the DAT mutations impair the dynamic complexity of striatal DA release patterns, that, in turn, may limit its informational value. We also performed a cross-correlation analysis to examine the temporal coordination of DA signals between DS and VS. In agreement with our earlier observations in WT mice (42), we observed a sharp, asymmetric peak with the DS signal preceding the VS DA signal by about 0.35 seconds, supporting a highly temporally structured and directional relationship between these regions. DAT-I312F/D421N^+/+^ mice, however, exhibited a markedly different temporal coordination, evident as a prolonged and less defined correlation pattern (although a peak still was seen at ~0.35 seconds). Thus, the precise temporal coupling between DS and VS DA release events appeared lost and replaced by more diffuse waves of DA that unfolded over longer times.

**Figure 9:**
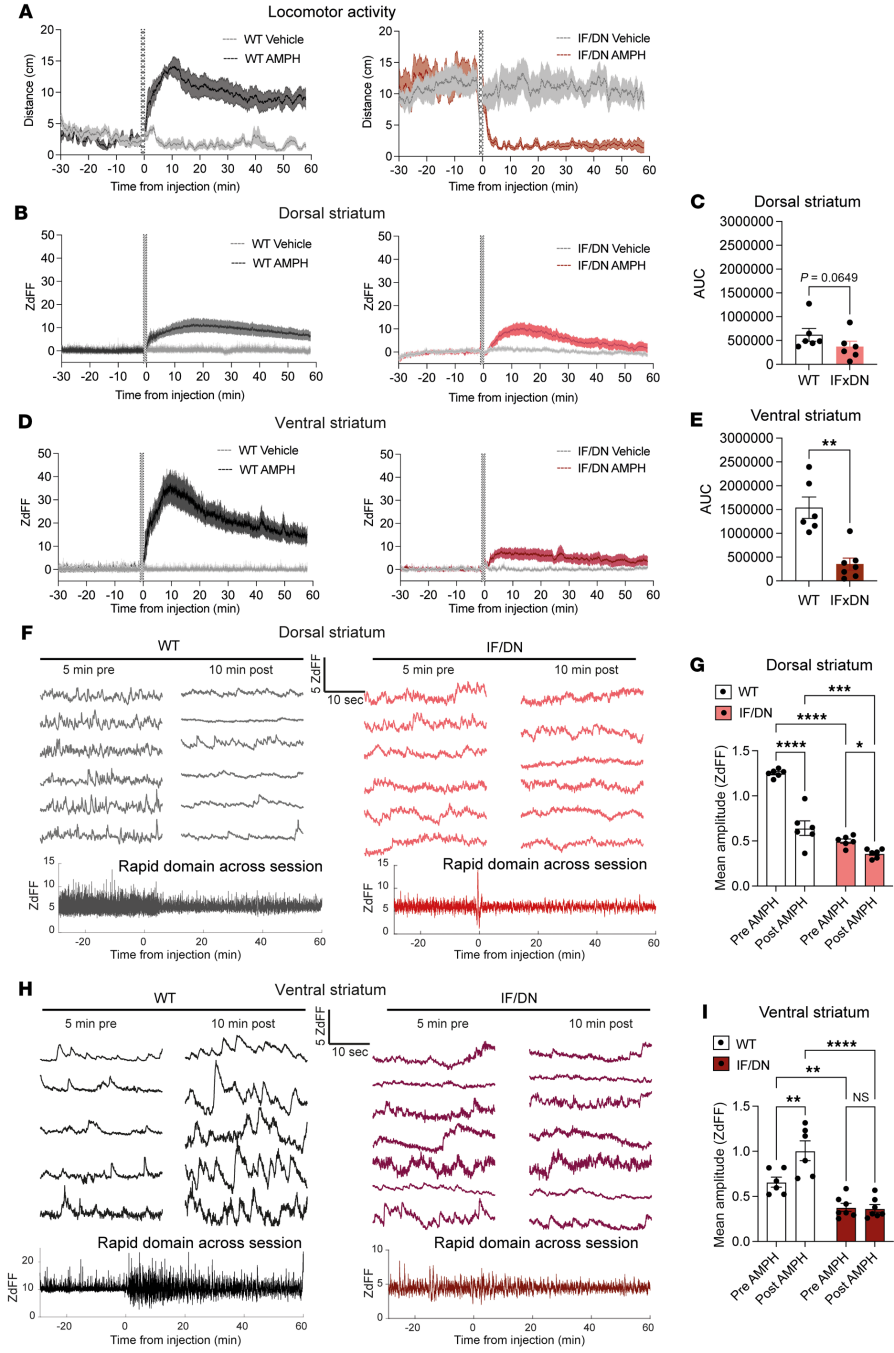
Region-specific disruption of AMPH-evoked DA release and fast DA dynamics in DAT-I312F/D421N^+/+^ mice. (**A**) Locomotor activity in 1-second time bins for WT and DAT-I312F/D421N^+/+^ mice during in vivo recording of DA signals before and after AMPH (2 mg/kg, i.p.) administration. (**B**–**E**) Average saline- and AMPH-induced DA responses in WT (left) and IF/DN (right) mice measured in DS (**B** and **C**) and VS (**D** and **E**). Area under the curve (AUC) following AMPH administration shows no significant genotype difference in DS (**C**, *P* = 0.065, unpaired *t* test. *N*_AMPH_ = 6 per genotype) but a marked reduction in AMPH-evoked DA release in VS of IF/DN mice (**E**, *P* < 0.01, unpaired *t* test. *N*_AMPH_ = 6 WT and 6 IF/DN mice). (**F**–**I**) Analysis of AMPH’s impact on fast DA dynamics. **F** and **H** show representative 30-second DA traces from WT and IF/DN mice in DS and VS, respectively, alongside representative examples of the rapid signal component across an entire recording session (band-pass filtering for rapid fluctuations: 0.01–10 Hz). **G** and **I** show the quantification of mean signal amplitude of the rapid DA signal component before and after AMPH injection. In WT mice, AMPH reduces rapid signal amplitude in DS (**F** and **G**) but increases the mean amplitude in VS (**H** and **I**), whereas in IF/DN mice AMPH produces only a modest increase in DS and no significant change in VS (2-way ANOVA with Holm-Šidák correction. *N* = 6 WT and 6 DS or 7 VS IF/DN mice). Data are shown as mean ± SEM. *P < 0.05, **P < 0.01, ***P < 0.001, ****P < 0.0001.

Upon AMPH administration, we observed an expected increased locomotor activity in the WT mice, which was accompanied by a large increase in striatal DA (Figure 9, A–E). Notably, the magnitude of the DA response was substantially greater in the VS than in the DS (~35 times SD of baseline in VS versus ~10 times SD of baseline in DS; Figure 9). In the DAT-I312F/D421N^+/+^ mice, locomotor activity was again markedly reduced by AMPH. Strikingly, this hypolocomotion was not accompanied by a significant change in the overall AMPH-induced DA response in DS compared with WT mice (*P* = 0.065, quantified by the AUC; Figure 9, B and C). In VS, however, AMPH-evoked DA elevation was profoundly impaired (Figure 9, D and E). Finally, we evaluated AMPH’s effect on fast DA dynamics. Visual inspection of representative 30-second traces before and after AMPH administration revealed a marked blunting of rapid DA dynamics in DS of WT mice with reduced signal amplitude (Figure 9, F and G), whereas signal amplitudes in the VS were increased (Figure 9, H and I). These observations were supported by isolating the fast (0.01–10 Hz) frequency components of the DA signals across the entire 90-minute recording periods and quantifying mean amplitudes before and after AMPH administration (Figure 9, G and I). In contrast, DAT-I312F/D421N^+/+^ mice showed no apparent change in VS dynamics and only a modest, yet significant, effect in DS (Figure 9, F–I).

Collectively, these in vivo recordings of AMPH-induced DA responses reveal a striking alteration in the regional specificity of AMPH-induced DA signaling in DAT-I312F/D421N^+/+^ mice, suggesting a fundamental shift in striatal DA regulation, which may underlie the opposite behavioral responses to AMPH observed in this model.

## Discussion

Atypical DTDS was recently described as a clinical entity distinct from classical DTDS with parkinsonian motor symptoms emerging in adolescence or adulthood rather than in infancy or early childhood. Strikingly, the motor symptoms in adult-onset atypical DTDS are preceded by neuropsychiatric disturbances (23, 24, 47), supporting putative shared underlying pathobiological pathways. Consequently, the investigation of DAT variants linked to atypical DTDS might not only provide key insights into the impact of DAT on DAergic neural mechanisms and how DAergic deficits are linked to disease but also advance our broader understanding of DA-related pathologies. Here, we present a construct-valid mouse model of atypical DTDS based on DAT variants identified in a patient with atypical DTDS exhibiting early-onset parkinsonism and ADHD (23). Our study demonstrates that the compound heterozygous genotype (DAT-I312F/D421N^+/+^) induces severe DAT dysfunction, which results in neurochemical disturbances that correlate with altered drug responses and psychomotor phenotypes, collectively recapitulating core features of atypical DTDS. DAT uptake capacity is reduced by approximately 75% in DAT-I312F/D421N^+/+^ mice, which drives a global disturbance of DA homeostasis characterized by elevated basal levels of extracellular DA, compromised DA release, and reduced DA tissue content. Importantly, immunohistochemical stainings show clear striatal DAT expression in DAT-I312F/D421N^+/+^ mice, suggesting that reduced catalytic activity underlies the loss of uptake function, consistent with prior heterologous cell data (23, 24).

Our analyses further demonstrated both pre- and postsynaptic adaptive changes, including reduced protein levels of TH and DA receptors (DR1 and DR2, Figures 2 and 4). It is possible that the elevated extracellular DA levels in DAT-I312F/D421N^+/+^ lead to a downregulation of postsynaptic DA receptors and reduce TH in DAergic neurons via increased tonic stimulation of presynaptic inhibitory DR2s. This is supported by the FSCV data demonstrating that blockage of DR2s with sulpiride produced a larger disinhibition of DA release from striatal slices from DAT-I312F/D421N^+/+^ mice compared with WT mice. Downregulation of TH could in turn exacerbate or act as a key driver of the intracellular DA deficiency, which is already compromised by the impaired DA reuptake via DAT and enhanced DR2-mediated autoinhibition of release. Of interest, the FSCV recordings also revealed region-specific effects of the mutations with more pronounced impairment of release and uptake kinetics in the DS than in the VS. This is particularly interesting as it has become increasingly clear that subdomain heterogeneity within the striatum affects behavioral outcomes through differential DAergic signaling (48, 49). The regional differences might be a consequence of DAT being expressed at higher levels in DAergic neurons from SN, projecting predominantly to DS, than in DAergic midbrain neurons that mainly target the VS (50). Accordingly, we conclude that the DS likely relies more heavily on DAT for maintaining intracellular DA stores and for DAT-dependent regulation of DA transients (51).

Overall, our investigations of the DA system dysfunction in DAT-I312F/D421N^+/+^ mice provide a complex picture of a pathology involving hyperdopaminergia in a global DA-deficient state with both pre- and postsynaptic plasticity. These findings underscore how disease-associated DAT dysfunction reshapes DA dynamics and translates into altered DA-regulated behaviors and drug responses, thereby resonating with earlier studies of genetically modified DAT mice (25, 52–55). It is interesting to note that the neurochemical profile of DAT-I312F/D421N^+/+^ mice, with approximately 25% residual DA uptake capacity, could be expected to represent an intermediate profile of homozygous and heterozygous DAT-KO mice. However, DAT-KO heterozygous mice with 50% uptake capacity show no or only discrete behavioral phenotypes (56, 57), while the DAT-I312F/D421N^+/+^ mice display clear changes in DA-regulated behaviors that recapitulate aspects of movement disorder and ADHD. This suggests that neural functions are greatly perturbed when DAT activity falls below 50% in mice. It is also likely that the distinct molecular phenotypes of the DAT-I312F and DAT-D421N mutants cause unique neurophysiological disturbances that act in concert with diminished DAT activity to exacerbate the DAergic dysfunction. Most notably, the DAT-D421N mutation is located in the second sodium binding site and introduces a sodium leak current and a bias toward an inward-facing conformation that supports constitutive DA efflux in vitro. The DAT-I312F mutation, on the other hand, changes the substrate-coupled anion conductance (23, 39). From a structural-functional perspective, the effect of the individual mutations on DAergic neuronal physiology would be particularly interesting to study in single-mutant knockin mice, as these cannot be separated in the compound heterozygous mice.

Genetic mouse models of familial forms of PD are limited by the general absence of clear motor impairments and DAergic neurodegeneration, unless additional genetic or exogenous strategies are employed to exacerbate the phenotype (35, 58). Moreover, although the underlying cellular mechanisms are likely model specific, several PD-relevant mouse models have also been reported to display early hyperlocomotion or increased extracellular DA, as we also observed in the DAT-I312F/D421N^+/+^ mice, particularly during presymptomatic or compensatory phases (59–62). In DAT-I312F/D421N^+/+^ mice, we further observed motor phenotypes, such as hind limb clasping and kyphosis, which could be indicative of beginning neurodegeneration. However, there was no evidence for DAergic cell loss or α-synuclein pathology. Yet the “dying back hypothesis” proposes that DAergic neurodegeneration is preceded by loss of axon terminals and has received attention as a potential early disease state in PD (63, 64). Consistent with this idea, we detected a reduction in area density of DAergic terminals in DAT-I312F/D421N^+/+^ mice, suggesting that DAT dysfunction may promote early axonal vulnerability. Although DA toxicity most often relates to the damaging effects of intracellular DA (65, 66), the elevated extracellular DA levels in the DAT-I312F/D421N^+/+^ mice might be directly implicated in the loss of DA terminals via DA toxicity (67, 68). Another intriguing possibility is that the increased extracellular DA levels in DAT-I312F/D421N^+/+^ mice drives the reduction in DAergic fiber density via presynaptic D2Rs, which have been shown to act as negative regulators of the DAergic axonal arbor (69–71). Furthermore, it is possible that the unique changes in electrophysiological properties of both DAT-I312F and DAT-D421N contribute to neuropathological processes (23, 39). Interestingly, our analysis of neonatal pups indicated that the reduction in fiber density is, at least partially, developmental. Such early alterations in the DAergic system might not only be a critical substrate for neuropsychiatric conditions but also present an increased vulnerability to age-related degeneration and environmental insults.

The DAT-I312F/D421N^+/+^ mice exhibited an explorative behavioral phenotype, manifested as hyperactivity, excessive rearing, and reduced preference for closed arms in the EPM. These findings support the hypothesis that the mutations drive aberrant ADHD-related behaviors as a consequence of the dysregulated DA neurotransmission. These behaviors may be linked to increased extracellular DA levels, which has previously been associated with heightened locomotor activity and exploratory behavior (52, 72). Our microdialysis experiments support this link, as we observed elevated basal DA levels in DAT-I312F/D421N^+/+^ mice. Yet the relationship between extracellular DA and spontaneous activity appears more complex than tonic levels alone, as AMPH paradoxically alleviates the hyperactive behaviors of DAT-I312F/D421N^+/+^ mice despite further increasing DA levels. Moreover, our in vivo recordings of striatal DA dynamics using dLight1.3b provided important additional insight into how the temporal structure and regional coordination of DA signaling are perturbed in freely moving DAT-I312F/D421N^+/+^ mice. In WT mice, we observed distinct regional patterns during self-paced exploration: rapid, high-frequency transients in DS and slower, more integrative fluctuations in VS, consistent with the idea that regional specialization in DA dynamics supports complementary behavioral functions (42–44, 73). In DAT-I312F/D421N^+/+^ mice, however, fast DA fluctuations were nearly absent, replaced by broad, irregular waves in both DS and VS. Spectral analysis confirmed a marked loss of high-frequency activity. Cross-correlation analysis further revealed a breakdown in the precise temporal coordination between DS and VS, and first-derivative variance analyses indicated a significant reduction in signal complexity. These changes in DA dynamics are likely to reduce the informational value of the DA signal and impair striatal information processing. Interestingly, the changes did not result in any major impairment of motor functions beyond clasping in the DAT-I312F/D421N^+/+^ mice; however, it is conceivable that the changes might be linked to the “uninhibited” hyperactive and explorative phenotype. It is also tempting to speculate that such alterations might have consequences for more complex behaviors. Future studies should further clarify this interesting issue. As for AMPH, we observed, somewhat surprisingly, that the overall AMPH-induced DA response was largely preserved in the DS of mutant mice yet profoundly blunted in the VS. This regional decoupling parallels the behavioral dissociation with reduced locomotor responses despite AMPH-induced elevated extracellular DA and suggests that the therapeutic versus psychostimulant actions of AMPH may critically depend on dynamic, VS-specific DA reactivity or the balance between dorsal and ventral striatal DA release. Of note, the generally preserved DS response to AMPH differs from the markedly reduced response observed in our microdialysis experiments. An appealing explanation for this apparent discrepancy is that movement-related DA activity in the DS of the freely moving mice in the fiber photometry experiments can partially sustain the responses to AMPH while this is not the case for the anesthetized mice in the microdialysis experiments. Taken together, our findings support that DAT is central for both temporal signal fidelity and regional specialization in striatal DA transmission (74).

The aberrant drug responses in DAT-I312F/D421N^+/+^ mice were not restricted to drugs that target DAT. ORPH, a drug with a mostly anticholinergic effect, also alleviated the hyperactive behavior of DAT-I312F/D421N^+/+^ mice in an open-field arena. This observation holds translational relevance, as the patient carrying the 2 mutations shows a positive clinical response to ORPH. It also suggests that a neurochemical dysfunction that originates in DAergic neurons can cause more widespread circuit disturbances, which in turn alter cholinergic responses (40, 41). Future work should aim at uncovering cellular and circuit deficits in other neurotransmitter systems in these mice and elucidate how such deficits relate to DAT-dependent DA dysfunction. In this way, the patient-based DAT-I312F/D421N^+/+^ mouse model generated in this study might not only represent a construct-valid model of atypical DTDS but also serve as an important tool for dissecting complex neurobiological interactions that can inform about DA-related diseases and potentially even guide new avenues for their treatment.

## Methods

### Sex as a biological variable.

Both male and female mice were included to support broader generalizability. Apart from body weight comparisons (analyzed by sex, given known differences), sex was not a prespecified variable, and data were pooled to assess genotype effects.

### Generation, breeding, housing, and genotyping of DAT-I312F/D421N^+/+^ mice.

The compound heterozygous DAT-I312F/D421N^+/+^ mice were obtained by crossing heterozygous DAT-I312F^+/–^ females and male DAT-D421N^+/–^ single-mutant mouse lines, which were generated by genOway S.A. (Supplemental Methods and Supplemental Figure 1) (53). Mice in breeding cages were kept on a high-fat diet (Research Diets D12451), while offspring received regular chow food after weaning. All mice were kept on a 12-hour light/12-hour dark cycle with food and water available ad libitum. Primers for genotyping are listed in Supplemental Methods.

### Synaptosomal uptake.

DA uptake assays on synaptosomal preparations were carried out as described previously (75). All experiments were performed on pairs of WT and DAT-I312F/D421N^+/+^ mice. All pairs were independently processed and matched WT and DAT-I312F/D421N^+/+^ samples were run in parallel during the entire experiment. A 2-fold dilution row with constant specific activity (final DA concentrations 1–0.031 μM) containing a fixed-ratio mixture of unlabeled DA and 2, 5, 6-[^3^H]-DA (Perkin Elmer Life Sciences) was used to obtain saturation DA uptake curves, which were fitted with Michaelis-Menten kinetics to derive *V_max_* and *K_m_* values.

### HPLC analysis of total DA tissue content.

Mouse brains were dissected to obtain DA, VS, and midbrain samples, which were snap-frozen in liquid nitrogen immediately after dissection and kept at –80°C until processing. The tissue samples were homogenized using 300 μL of lysis buffer (20 mM HEPES, 125 mM NaCl, 10% glycerol, 1 mM EDTA, 1 mM EGTA) supplemented with 1% protease inhibitor cocktail (Millipore) and 30 μL 1N HClO_4_. The homogenate was centrifuged for 15 minutes at 16,000*g* (4°C), and the supernatant was collected and filtered through a 0.22 μm filter (MilliporeSigma). DA content was analyzed using high-performance liquid chromatography with electrochemical detection as previously described (76). DA content was normalized to the total protein in each sample.

### FSCV.

FSCV recordings were made on acute brain slices from adult DAT-I312F/D421N^+/+^ mice and WT littermates. The animals were anesthetized with halothane, then quickly decapitated, and the brain was harvested. Next, the brain was submersed in ice-cold oxygenated artificial cerebrospinal fluid (aCSF) containing in mM NaCl 125, KCl 2.5, KH_2_PO_4_ 0.3, NaHCO_3_ 26, glucose 10, CaCl_2_ 2.4, and MgSO_4_ 1.3, and coronal striatal brain slices of 300 μm thickness were prepared with a VT1000S vibrating blade microtome. Slices were transferred to oxygenated aCSF at room temperature and allowed to recover for at least 1 hour. Single-pulse (1 ms, 400 μA) evoked release of DA in DS and VS and the effect of sulpiride (5 μM, 15 minutes preincubation) on train-evoked DA overflow (10 Hz, 30 pulses of 1 ms, 400 μA) was recorded by FSCV as previously described (27).

### Western blotting and acute brain slice biotinylation.

Whole tissue lysates from midbrain or striatum and crude striatal synaptosomes were prepared as previously described (24, 54, 75). Samples from surface biotinylated acute brain slices were prepared as described in Supplemental Methods. After adjustments of protein concentrations, equal amounts of protein were analyzed by SDS-PAGE, and Western blotting for TH, DAT, VMAT2, DR1, DR2 was carried out as described before (23, 24) (see antibodies in Supplemental Table 4). For α-synuclein immunoblots (both total and p-S129), samples were preheated at 70°C for 30 minutes prior to SDS-PAGE. For p-S129 α-synuclein detection, membranes were additionally fixed after transfer in 0.4% paraformaldehyde/PBS at room temperature, washed in 0.1% Tween-TBS, and blocked for 2 hours in prewarmed (60°C) blocking buffer (2% PVP-40/0.1% Tween-TBS). All blots were developed by chemiluminescence following incubation with secondary HRP-conjugated antibodies (1:2,000), and an HRP-conjugated anti–β-actin antibody (1:40.000, Sigma) was used as loading control. Relative band intensities were quantified using Fiji software.

### Immunohistochemistry and stereology.

Fluorescent immunocytochemistry was performed on coronal sections of the striatum and midbrain from transcardially perfused mice (4% paraformaldehyde in 0.1 M PBS pH 7.4) as described before (24). WT and DAT-I312F/D421N^+/+^ derived slices were stained in pairs and imaged in parallel. Slices were incubated overnight (4°C) with primary antibodies (see Supplemental Table 4) against TH, DAT, VMAT2, DR1 (see ref. 29 for KO validation), and DR2 (see ref. 30 for KO validation), followed by incubation overnight at 4°C or for 3 hours at RT with secondary Alexa Fluor 488–, Alexa Fluor 568–, and Alexa Fluor 647–conjugated antibodies (1:400, Thermo Fisher Scientific). Sections were mounted on glass coverslips (Mentzel-Gläzer, 24 × 60 mm) using ProLong Gold antifade mounting medium (Invitrogen).

Stereological analysis was performed on DAB-stained TH^+^ neurons in the SN of systematically sampled midbrain sections, as described in Supplemental Methods.

### Fluorescence imaging and image analysis.

Wide-field fluorescence images of entire striatal and midbrain sections were acquired using a Slide Scanner Axio Scan.Z1 with a Plan-Apochromat 20×/0.8 objective (Zeiss), and confocal images were acquired on an LSM 510 for adult mice and an LSM 710 for neonatal mice with an oil immersion 63×/1.4 numerical aperture objective (Carl Zeiss) as described in Supplemental Methods.

Image processing and analysis of immunoreactivity and fiber area density was carried out using Fiji software (NIH ImageJ2) and detailed in Supplemental Methods.

### qPCR analysis.

Mouse brain tissue samples were obtained from age-matched male and female WT and DAT-I312F/D421N^+/+^ mice. mRNA levels from striatal and midbrain lysates were analyzed as previously described, using 1,000 ng of RNA from each sample for cDNA synthesis (77). Gene expression data are presented as relative expression, calculated as fold-change over the WT mean, using the 2^(-ΔΔCt)^ method (78). Gapdh and Hprt were used as housekeeping genes, as their expression levels were consistent across genotypes and brain regions. Primers are listed in Supplemental Table 5.

### Behavioral analysis.

Behavioral testing of DAT-I312F/D421N^+/+^ mice and WT littermates was conducted on adult mice over a period of 5 weeks starting at age 13–19 weeks. All experiments were conducted in the same order, and male and female mice were tested separately. Cage-changing was prohibited 3 days prior to experiments. Experiments were conducted during the light cycle, at the same time of the day (0930 to 1730), in a quiet room with indirect lighting, and mice were habituated to the test room for 45 minutes prior to experiments. Equipment was cleaned with 70% ethanol between each animal or trial. All behavioral test procedures are described in Supplemental Methods.

### Microdialysis.

Basal and AMPH-induced extracellular DA levels were measured in the DS using slow-flow in vivo microdialysis under anesthesia, followed by HPLC-based quantification. Methodological details are provided in Supplemental Methods.

### Stereotactic surgeries and in vivo fiber photometry.

Stereotactic injections of AAV9-hSyn-dLight1.3b-WPREpA and implantation of optical fibers into the DS and VS were performed as described previously (42) and in Supplemental Methods. Fiber photometric recordings of dLight1.3b fluorescence were performed on a Neurophotometrics FP3001 or FP3002 system as detailed in Supplemental Methods. Briefly, a multibranch fiber optic path cord (Doric Lenses, 200 μm, NA 0.37), was attached to the optic cannula implants, with bronze mating sleeves (Thorlabs). Fiber photometry recordings were performed using the open-source software Bonsai (79). Light power was adjusted to approximately 25 μW and 18 μW and for the 470 channel and isosbestic 415 nm channel, respectively (minor variation across patch cord branches). DA dynamics and AMPH-evoked responses (2 mg/kg, Sigma-Aldrich) were recorded in open-field arenas with AMPH or saline administered i.p after a 30-minute baseline period. Data were processed and analyzed using MATLAB (MathWorks) and Prism 10 (GraphPad Software) as detailed in Supplemental Methods.

### Statistics.

Prism 9.0 (GraphPad Software) software was used for statistical analysis and data fitting. All statistical methods are specified in the figure legends. Data are presented as mean ± SEM. Paired or unpaired (depending on the experimental design) 2-tailed *t* test was applied for 2-group comparisons for normally distributed data. Mann-Whitney *U* test (unpaired) or Wilcoxon’s signed-rank test (paired) was used for data that failed normality tests. A 2-way ANOVA with Holm-Šidák posttest was used for multiple comparisons. Differences were considered significant at *P* values lower than 0.05.

### Study approval.

All animal experiments were approved by the Danish Animal Experimentation Inspectorate (permission numbers 2017-15-0201-01160; 2017-15-0201-01177; and 2022-15-0201-01216), and by local authorities, and adhere to the European guidelines for the care and use of laboratory animals, EU directive 2010/63/EU.

### Data availability.

Custom written code used for processing of fiberphotometry data is available at https://github.com/GetherLab/DAT-I312F-DAT-D421N, commit ID cb4ae144f0f78d2975329d0fc6a517a5a4181d2f. Source data for all graphs are provided in the Supporting Data Values file.

## Author contributions

FH and UG conceptualized the study. FH, LKK, SHJ, JHL, CFP, BDL, FPM, SAB, LPP, CFR, NA, JAP, and FB conducted the experiments. FH, UG, MR, GT, LET, BH, ALE, MVO, SA, and DW provided supervision. FH and UG prepared the final figures and wrote the original manuscript. FH and UG provided funding. All authors contributed to the editing and review of the manuscript.

## Funding support

- Independent Research Fund Denmark – Medical Sciences (DFF-4183-00571, FH).
- Independent Research Fund Denmark – Medical Sciences (DFF 4004-00097B, UG).
- The Lundbeck Foundation (R181-2014-3090 and R303-2018-3540, FH).
- The Lundbeck Foundation (R223-2016-261, R266-2017-4331, R276-2018-792, and R359-2020-2301, UG).
- The Novo Nordisk Foundation (NNF24OC0088870, FH).
- Fondecyt Initiation Fund (11191049, JAP).
- Canadian Institutes of Health Research (PJT-165928 and PJT-183931, LT).
- The Krembil Foundation (LT).

## Supplementary Material

Supplemental data

Unedited blot and gel images

Supplemental video 1

Supplemental video 2

Supplemental video 3

Supplemental video 4

Supplemental video 5

Supplemental video 6

Supporting data values

## Version 1 01/27/2026

In-Press Preview

## Version 2 03/16/2026

Electronic publication
