# Supplementary material for: Mouse model of atypical DAT deficiency syndrome uncovers dopamine dysfunction associated with parkinsonism and ADHD
Source: J Clin Invest. 2026 Jan 27;136(6):e169297. doi: 10.1172/JCI169297 (PMC12987629; doi:10.1172/JCI169297)
Supplement: Supplemental data [file jci-136-169297-s041.pdf]

Supplementary information

Mouse model of atypical DAT deficiency syndrome uncovers dopamine dysfunction associated with parkinsonism and ADHD

Freja Herborg[1*], Lisa K. Konrad[1], Søren H. Jørgensen[1], Jamila H. Lilja[1], Benoît Delignat-Lavaud[2], Leonie P. Posselt[1], Ciara F. Pugh[1], Sofie A. Bach[1,3], Cecilia F. Ratner[4], Nora Awadallah[5], Jose A. Pino[6], Frida Berlin[1], Aske L. Ejdrup[1], Mikkel V. Olesen[3], Mattias Rickhag[1,7], Birgitte Holst[4], Susana Aznar[3], Felix P. Mayer[1], David Woldbye[1], Gonzalo Torres[5,8], Louis-Eric Trudeau[2], Ulrik Gether[1*]

[1]Molecular Neuropharmacology and Genetics Laboratory, Department of Neuroscience, Faculty of Health and Medical Sciences, University of Copenhagen, Copenhagen, Denmark.
[2] CNS Research Group, Department of Pharmacology and Physiology, Department of Neurosciences, Faculty of Medicine, Université de Montréal, Montreal, QC, Canada.
[3]Centre for Neuroscience and Stereology, Copenhagen University Hospital Bispebjerg-Frederiksberg, Denmark.
[4] Department of Biomedical Sciences, University of Copenhagen, Copenhagen, Denmark.
[5] Department of Molecular, Cellular, and Biomedical Sciences, City University of New York School of Medicine at City College, New York, United States.
[6] Laboratorio de Bioquímica y Farmacología Molecular, Escuela de Ciencias, Facultad de Ciencias de la Vida, Universidad Viña del Mar.
[7]Danish Research Centre for Magnetic Resonance (DRCMR), Department of Radiology and Nuclear Medicine, Copenhagen University Hospital - Amager and Hvidovre, Copenhagen, Denmark
[8] Department of Molecular Pharmacology and Neuroscience, Stritch School of Medicine, Loyola University, Chicago, United States.

[*]Address author correspondence to: Freja Herborg, Department of Neuroscience, Maersk Tower 7.5, University of Copenhagen, Blegdamsvej 3B, DK-2200 N, Copenhagen, Denmark. Phone +4553609699; E-mail: frejahh@sund.ku.dk. Or to: Ulrik Gether, Department of Neuroscience, Maersk Tower 7.5, University of Copenhagen, Blegdamsvej 3B, DK-2200 N, Copenhagen, Denmark. Phone +45 2875 7548; E-mail: gether@sund.ku.dk.

SUPPLEMENTARY MATERIALS AND METHODS

Generation of DAT-I312F/D421N$^{+/+}$ mice

The compound heterozygous DAT-I312F/D421N$^{+/+}$ mice were generated by crossing heterozygous DAT-I312F and DAT-D421N single-mutant lines (Supplementary Figure 1). Both knock-in strains were custom-generated by GenOway S.A. (Lyon, France) using classical homologous recombination in C57BL/6 embryonic stem (ES) cells and genomic homology arms derived from isogenic C57BL/6 DNA to introduce the hDAT-I312F (mDAT-I311F) and the hDAT-D421N mutation (mDAT-D420N) in the murine *Slc6a3* gene for expression under the control of the endogenous *Slc6a3* promoter. To generate the DAT-D421N line (corresponding to mDAT-D420N), a targeting vector was constructed using C57BL/6 genomic DNA and contained a long homology arm of ~4.8 kb spanning exons 10–13 and neighbouring intronic regions, and a short homology arm of ~3.2 kb spanning exon 9 where the D420N point mutation (GAC→AAC) was introduced. A loxP-flanked neomycin resistance cassette was inserted into intron 9 for positive selection. The targeting vector to generate the DAT-I312F line (corresponding to mDAT-I311F), was likewise constructed using C57BL/6 genomic DNA and contained a long homology arm of ~5.2 kb spanning exons 5–6 with neighbouring intronic regions, and a short homology arm of ~2.0 kb spanning exon 7 where the I311F point mutation (ATC→TTC) was introduced. The loxP-flanked neomycin resistance cassette for positive selection was inserted into intron 6. Both targeting vectors were validated by sequencing and restriction analysis. Following linearization by restriction digest, each targeting vector was electroporated into ES cells derived from black-coated C57BL/6J mice. Positive selection (200µg/ml G418) was started 48 hours after electroporation. Correctly targeted ES cell clones were identified by PCR screening, direct sequencing, and Southern blot verification of homologous recombination event at both 5' and 3' ends. Recombined ES cell clones, (from black-coated C57BL/6 mice), were injected into blastocysts, derived from an albino C57BL/6J mouse strain and re-implanted into pseudopregnant females to produce chimeras. Chimeras were identified by coat color and male chimeras (>50%) were bred with Cre-recombinase expressing C57BL/6 mice to excise the *lox*P flanked neomycin cassette. Resulting heterozygous mice carrying the neo-excised point mutant knock-in allele were validated by PCR genotyping and by Southern blotting to confirm successful recombinase-mediated excision of the neomycin cassette.

Genotyping of DAT-I312F/D421N$^{+/+}$ mice

Ear or tail biopsies were used for PCR genotyping using the following primers for detection of the I312F mutation: 5' - CTAGGGATGCGGTAGGGCTTGTTTC - 3' and 5' - CTGGGGATGCTTCATTTCCAAGAGG - 3', and for detection of the D421N mutation: 5' - GGACATGAGTGTTAGTGGCTGGAATGTAAC - 3' and 5' - CTCAGTAAATAATCTTGAGCAGTTGATGGGG - 3'.

Acute brain slice biotinylation assay

The assay was performed based on previously published protocols (1, 2) under constant oxygenation (95% O_2/5% CO_2). Pairs consisting of one WT and one DAT-I312F/D421N$^{+/+}$ mouse were humanely euthanized and brains were rapidly removed before transfer into ice-cold sucrose-artificial cerebrospinal fluid (S-aCSF; sucrose 250, KCl 2.5, NaH2PO4 1.2, NaHCO3 26, D-glucose 11, MgCl2.6H2O 1.2, CaCl2.2H2O 2.4, all in mM pH 7.4). Subsequently, a Leica VT100S vibratome was used to collect 290 µm thick coronal slices containing the ventral and/or dorsal striatum (AP +0.2 mm to AP+1.5 mm, relative to Bregma) in ice-cold S-aCSF. Next, the slices were transferred into aCSF (NaCl 124, KCl 2.5, NaH2PO4 1.2, NaHCO3 26, D-glucose 11, MgCl2*6H2O 1.2, CaCl2*2H2O 2.4, all in mM, pH 7.4) and recovered at 32°C for 45min. Following recovery, the slices were washed 3 times with ice-cold aCSF and subjected to membrane-impermeable EZ-Link™ Sulfo-NHS-Biotin (Thermo Fisher) (1 mg/ml) for 30 min on ice. The reaction was quenched by exposing the slices to 100mM glycine for 20 min (replacement of the glycine solution after 10 min). Following three rapid and two 5-min washes, the VS and DS were dissected, and the tissue was stored at -80 °C until processed. The tissue was solubilized in RIPA buffer containing protease inhibitors (Roche cOmplete™ Protease Inhibitor Cocktail). The protein concentration of the lysates was determined using the BCA protein assay (ThermoFisher). Biotinylated proteins were extracted by exposing 20 µg of total protein to 50 µL streptavidin agarose resin slurry (Pierce™ Streptavidin Agarose) in a final volume of 600 µL for a minimum of 4 h at 4 °C under constant over-head rotation. 20 ug of protein were set aside in a final volume of 150 µL. Following 3 washes with RIPA buffer containing protease inhibitors, the protein bound to the streptavidin agarose resin was denatured/eluted with Laemmli Sample Buffer (Bio-Rad; supplemented with β-mercaptoethanol) at room temperature for 30 min. Surface and total protein were finally separated by SDS-PAGE, transferred onto PVDF membranes, followed by immunoblotting (see section on western blotting in main text for details).

Fluorescent imaging and image analysis

Wide field imaging of brain slices and intensity analysis

Wide-field fluorescent images of entire striatal and midbrain sections were acquired using a Slide Scanner Axio Scan.Z1 with a Plan-Apochromat 20x/0.8 objective (Zeiss). Alexa-488, Alexa-568, and Alexa-647 emissions were recorded using a 525/50 nm band pass (Alexa 488), 605/70 nm band pass (Alexa 568) and 690/50 nm band pass (Alexa 647) filters with an identical exposure setting for WT and DAT-I312F/D421N$^{+/+}$ mice. Image processing and analysis of immunoreactivity were carried out using Fiji software (NIH ImageJ2). Nonspecific signal was subtracted from each image and rectangular ROIs around the entire midbrain or bilateral striatae were defined. Automated threshold functions were applied for unbiased isolation of labelled structures ('Li threshold' function for striatal images, 'mean threshold' for TH midbrain and 'default threshold' function for DAT and VMAT2 midbrain stains). The mean signal intensity of the threshold area was measured within each ROI and used to compare the signal intensities in WT and DAT-I312F/D421N$^{+/+}$ sections

Confocal imaging of striatal brain slices from adult mice and neonatal pups

Confocal images of DAT and TH immunoreactivity were used for analysis of DAergic terminal area density using a line-scan intensity analysis, modified from (3). Importantly, this analysis accounts for potential differences in protein expression (as seen for TH), as it employs a Hessian matrix that adjusts for differences in labelling intensity. Confocal images were acquired on a LSM 510 for adult mice and a LSM710 for neonatal mice with an oil immersion 63x/1.4 numerical aperture objective (Carl Zeiss, Oberkochen, Germany). Alexa-488 dye was excited using an argon-krypton laser and the emitted light was detected using a 505-530-nm band pass for adult mice, and a detection wavelength range of 494.5-572.2 nm for neonatal mice. A 543-nm or 633-nm helium-neon laser was used to excite the Alexa-568 or Alexa-647 fluorophores, respectively. Fluorescence was obtained using a 560-nm long-pass filter for adult mice or using a detection wavelength range of 630.0-758.5 nm for neonatal mice. For adult mice, three WT:IF/DN pairs were stained in parallel on the same day, and identical settings for matched WT:IF/DN pairs were used during imaging which was done blinded to genotype. For neonatal mice (P2-P5), all brains (five per genotype) were stained in parallel, and imaging was performed blinded to genotype using the same settings for all brains. Images with regional comparability were systematically collected throughout each striatal slice. 32 or 16 non-overlapping images were acquired from each mouse in the adult and neonatal mice, respectively.

Striatal fiber area density analysis

To quantify fiber area density from confocal images of dopaminergic projections in striatum of WT and DAT-I312F/D421N$^{+/+}$ mice, we implemented a line-scan intensity analysis inspired by (3). To minimise the risk that potential differences in terminal density reflected differences protein expression, rather than true terminal loss, all images were first fitted with a Hessian-based image filter in Image J (version 2.0.0, plugin, Feature J (4). This filter uses the Hessian matrix to find local intensity variations – i.e. maxima (fluorescent fibers) and minima (background) - and enhance their extremities to extract the curvilinear structures of the labelled fibers. The images were then converted to a binary image for subsequent analysis of fiber density. When converting images, "smoothing scale factor" was set to 0.5 and the "absolute eigenvalue comparison" option was chosen, generating a resulting image of largest eigenvalues (extrema) – settings were chosen in conjunction with (3). A line-scan intensity analysis was then carried out on each image using a grid of vertical and horizontal lines of same length (202.031μm for adult mice and 125.835μm for neonatal mice) and equidistant apart (lines created conformed to a set grid function in ImageJ) for an unbiased and automated analysis of the number of fibers crossing each line (Supplementary Figure 4). The pixel intensity along each line was plotted using ImageJ's "Plot Profile" tool (Supplementary Figure 4D). Only lines fully contained within the striatal region were profiled. Next, the line intensity profile data were imported to MATLAB (version 2018b 9.5.0.944444; MathWorks). Line scans were baseline-adjusted using the "msbackadj" command from MATLAB's bioinformatics toolbox. This algorithm determines the baseline by classifying data points into peaks and background, with the mean value of the background class used as the estimated baseline for each line scan. The resulting intensity matrices with adjusted baselines were then processed through a peak detection algorithm; "mspeaks" (Supplementary Figure 5). More detailed information on both processing commands is available in (5). For analysis of terminal density in the adult mice, the average background intensity from all line scans over the entire striatum between stained WT/KI pairs were used as a height/threshold filter. For terminal density analysis in the neonatal mice, the average background intensity for individual images were used as the height/threshold filter. Local maxima above the threshold were counted as peaks and used to represent an individual fibres crossing the line scan. The average number of peaks per line scan in each image was divided by the length of each line (202.031μm for adult mice and 125.835μm for neonatal mice) to yield the mean

value of peaks/fibers per µm. The results for WT and DAT-I312F/D421N$^{+/+}$ mice were then compared.

Stereological analysis

A quantitative analysis of the total number of TH-positive neurons in the substantia nigra (SN) from DAT-I312F/D421N$^{+/+}$ and control mice was performed using systematic uniform random sampling (SURS) and the optical dissector (6). Every 4th 40 µm-thick section was stained with a TH antibody using colorimetric free-floating immunohistochemistry according to a standard procedure. Briefly, sections were pretreated with 3% H2O2, heated to 92 °C in 10% Target Retrieval Solution (Agilent (Dako), USA) in PBS (phosphate-buffered saline) at pH 6, and incubated with 10% fetal calf serum in PBS. For labeling of dopaminergic neurons, the sections were incubated with TH antibody (1:90.000, AB152, Millipore), which was detected by Dako EnVision+ System-HRP as described by the manufacturer. The immunoreactions were visualized using 0.05% 3,3′-diaminobenzidine. Sections were counterstained using Cresyl Violet (Sigma-Aldrich, USA) followed by dehydration and mounting of coverslips. Stereological counting of cell numbers and volume estimations was performed using Stereo Investigator version 2021.1.3 (MicroBrightField Bioscience, USA) and an Olympus BX60 light microscope equipped with a Lumina HR camera (MicroBrightField Bioscience, USA). The precision of total cell number and volume estimates was provided as the coefficient of error (CE) according to (7) (see Supplementary Table 2 and 3). The sampling is considered optimal when CE is approximately half or less of the observed CV (= standard deviation/mean), which was the case in this study. To estimate the volume of a region, the Cavalieri principle was used (6).

Microdialysis

Mice were pretreated with the analgesic carprofen (5 mg/kg in 10 mL/kg, s.c.; Pfizer, New York, NY), anesthetized with isoflurane (induction ~4%, maintenance ~1%; Baxter, Deerfield, IL), and placed in a stereotaxic frame (Kopf Instruments, Tujunga, CA). A microdialysis probe (CMA7, 2 mm; CMA Microdialysis, Kista, Sweden) was inserted into the DS at bregma coordinates: AP, 1.2 mm; ML, ±1.5 mm; and DV, −4 mm (tip position relative to skull). The probe was connected to a syringe pump and perfused with artificial cerebrospinal fluid (aCSF; Harvard Apparatus, Holliston, MA). Microdialysis was performed in anesthetized mice at a flow rate of 1.2 µL/min. Microdialysates were collected every 10 min and analyzed immediately for DA content on an

HTEC-500 HPLC (Eicom, Kyoto, Japan), separated on a PP-ODS affinity column (Eicom), and electrochemically detected. Chromatograms were analyzed using EPC-300 software. After 40 min of stable baseline measurements, AMPH (2 mg/kg) was injected i.p., and microdialysates were collected for another 90 min. DA standards (0.5 pg/µL and 10 pg/µL) were run before and after microdialysate measurements. Relative DA levels (AUC normalized to the WT mean baseline) were used to depict the time course of AMPH-induced DA release and to compare relative baseline DA levels in WT and DAT-I312F/D421N$^{+/+}$ mice. For each mouse, the baseline DA level was determined as the mean of the four samples preceding AMPH administration. AMPH-induced DA efflux was quantified by DA peak levels expressed as the fold increase above its own baseline.

Behavioral analysis of DAT-I312F/D421N$^{+/+}$ mice

Behavioral testing of DAT-I312F/D421N+/+ mice and WT littermates was conducted in adult mice over a period of 5 weeks, starting at 15 ± 2 weeks of age. All experiments were conducted in the same order, and male and female mice were tested separately. Cage changing was prohibited for 3 days prior to testing. Experiments were conducted during the light cycle, at the same time of day (09:30–17:30), in a quiet room with indirect lighting, and mice were habituated to the test room for 45 minutes prior to testing. Equipment was cleaned with 70% ethanol between each animal and each trial.

Open field testing

Open field tests were used to evaluate spontaneous horizontal activity. Mice were placed in white open-field arenas (41 × 41 cm area; 50 cm height) and allowed to explore freely while being video-recorded from above. Tracking and analysis were performed in EthoVision XT (Noldus), with the center zone defined as the inner 20.5 × 20.5 cm^2. Locomotor responses to D-amphetamine (2–10 mg/kg; Sigma-Aldrich, #A5880), orphenadrine (30 mg/kg; Sigma-Aldrich, #04630), or saline were obtained by i.p. administration. For D-amphetamine at 2 mg/kg (and corresponding saline), injections were given after a 90-min habituation period in the open field. For D-amphetamine at 5 or 10 mg/kg, and orphenadrine (30 mg/kg) with their corresponding saline controls, injections were administered immediately prior to placement into the open field.

Elevated plus-maze

The elevated plus maze (arm length 35 cm, arm width 5 cm, wall height 15 cm; 50 cm above the ground) was used to assess exploratory versus anxiety-like behaviors. Prior to testing, mice were habituated to the experimenter and the test room by individually handling each mouse for 5 min on 3 consecutive days. On day 4, mice were placed on an open arm facing the center zone and video-recorded from above for 15 min. Tracking and analysis were performed in EthoVision XT to derive distance traveled, time spent in the open and closed arms (center point and tail point), and time spent in the center zone (center point). In addition, time spent nose-dipping from within the center zone or on the open arms was quantified as an additional measure of exploratory behavior.

Rearing

Mice were placed in an empty glass beaker (diam. 20 cm, height 22 cm) in front of two vertical mirrors arranged at an angle of approximately 110° and video recorded for 10 min. Frequency of rears, defined as lifting the front paws from the ground and upwards stretching the body were manually counted.

Activity measures in the home cage environment

For home cage activity recordings, mice were single housed in metabolic cages (Phenomaster, TSE systems, Bad Homburg, Germany) with food and water ad libitum. Nesting material could not be provided as infrared sensors were used to detect movement. Horizontal locomotor activity, grooming/fine activity, and rearing were monitored by infrared beam breaks. Measurements were collected for 8.5 days without human intervention except for required visual inspections.

Clasping and kyphosis

Clasping behavior was assessed by lifting each mouse by its tail during video recording. Videos of 30 s were analyzed manually at low speed in 3 s time-bins (i.e., 10 time-bins per video) for clasping behaviors. For each time-bin, a score of 0 was given if no clasping behavior was seen, 1 if the mice performed a 'half clasp' (one hind paw fully retracted to body midline or both hind paws retracted halfway to the body midline) and 3 if a clasp was fully established (both hind paws retracted, see Figure 6). The scores for each of the 10 time-bins were combined into a clasping score (hence the max score is 30).

To assess presence of abnormal anteflexion of the spine (kyphosis), mice were placed on a straight surface and observed with video recording during exploration for 60s. Kyphosis was scored as 0=not present, 1=mildly present and can be stretched out, 2=obviously present and cannot be stretched out.

Hanging Wire Test

To test muscle strength and coordination, mice were subjected to the hanging wire test (8). Mice were placed hanging by their forepaws in the middle of a fixed horizontal metal wire (2 mm thick and 40 cm long), positioned 37 cm above a cage filled with cushioning bedding, with escape platforms at both ends of the wire. All mice were video-recorded during up to 3 trials (intertrial interval, 10 min), with a maximum trial length of 300 s. Performance was scored manually as follows: 0 = fell off; 1 = hung onto the wire with two forepaws; 2 = hung onto the wire with an attempt to climb; 3 = hung onto the wire with two forepaws and one or both hind paws; 4 = hung onto the wire with all four paws and the tail wrapped around the wire; 5 = gripped the wire with all four paws and the tail wrapped around the wire and escaped to the platform; −1 = improper behavior. Improper behavior, such as balancing on or deliberately letting go of the wire, was addressed by immediately placing the mouse back on the wire. In addition to escape strategy scoring, we analyzed time on the wire. If the mouse fell from the wire, the experiment was repeated for a maximum of 3 trials, and the best score was used for statistical analysis.

Pole Test

To assess agility, coordination, and the ability to descend headfirst, we performed the pole test. Animals were trained for 5 trials on 2 consecutive days and tested with video recording for 5 trials on the third day. A wooden pole wrapped in gauze (50 cm long, 1 cm in diameter) was placed in the center of the home cage, with the base covered in bedding material. Mice were placed at the top of the pole with the head facing upward, and the time to descend was measured. The median across 5 trials (from video recordings) was used for analysis.

Rotarod

An accelerating rotarod (Panlab/Harvard Apparatus, USA; rod diameter 3.5 cm) was used to assess motor learning and coordination. Mice were first trained to stay on the rotating rod for 180 s at 5 rpm. Mice were then tested using an accelerating protocol (4–40 rpm over 5 min) for 5 consecutive days with 3 trials per day (intertrial interval, 10 min). The latency to fall was recorded, and the trial

was also stopped if a mouse completed two passive rotations. The best performance each day was used for statistical analysis

AAV injection and cannula implantation

Stereotactic surgeries were carried out as described in (9). riefly, mice were anesthetized using isoflurane (3–4% in 0.5% oxygen for induction; 1.5–2% for maintenance) and given a subdermal bolus injection of lidocaine hydrochloride at the incision site for local anesthesia. A Neurostar Stereotaxic Robot frame was used to inject 300 nL of AAV9-hSyn-dLight1.3b-WPREpA, diluted to a titer of 3.0 × 10^12 viral genomes/mL in sterile saline, into the DS at coordinates AP: 1.18 mm, ML: 1.70 mm, DV: −3.00 mm and into the VS at coordinates AP: 1.54 mm, ML: −0.80 mm, DV: −4.30 mm. An additional 100 nL was injected 200 µm below and 200 µm above the target coordinate. A metal ferrule optical cannula (200 µm core, 0.37 NA, 1.25 mm; Doric Lenses) was subsequently inserted at the target coordinate. Injection and implant sites (DS and VS) used for fiber photometry were calculated relative to bregma and lambda using Neurostar software. Implants were secured with Optibond™ FL sealing primer, Optibond™ FL Adhesive (Kerr™), and light-curing Tetric EvoFlow (Ivoclar Vivadent) dental cement. Incisions were closed with absorbable Vicryl suture (Ethicon). To prevent postoperative pain and infection, mice were treated with an analgesic/antibiotic mixture of carprofen (Rimadyl) and enrofloxacin (Baytril) in saline for 3 days. Mice were tested ≥3 weeks after surgery.

In vivo fiber photometry recordings

Fiber photometry recordings of dLight1.3b fluorescence in DAT-I312F/D421N$^{+/+}$ mice and WT littermates were performed using either a Neurophotometrics FP3001 or FP3002 system. For all recordings, a 3-m multibranch fiber-optic patch cord (Doric Lenses; 200 µm core, NA 0.37) was attached to the implanted optical cannulae using bronze mating sleeves (Thorlabs ADAL 4-5). Fiber photometry recordings were performed using the open-source software Bonsai (10).

Light power was measured at the patch cord tip under constant illumination using a power meter (Thorlabs PM100D with an S130C 400–1100 nm sensor) and adjusted to ~25 µW (minor variation across patch cord branches) for the 470-nm channel and ~18 µW for the isosbestic 415-nm channel. All recordings were performed in custom-made, bottomless white arenas (50 × 50 × 40 cm) from the local technical workshop. These were placed on a suspended glass plate to allow video recording from below. A Basler camera (model ACA1920-155UC) with a 6-mm F1.8 VS Technology lens (VS-0618H1) was used for recording. Center-point mouse movement was tracked

in Bonsai simultaneously with the fiber photometry recordings. DA dynamics and AMPH-evoked responses (2 mg/kg; Sigma-Aldrich) were recorded in the open-field arenas, with AMPH or saline administered i.p. after a 30-min baseline period. Data were processed and analyzed using MATLAB (MathWorks) and GraphPad Prism 10 (GraphPad Software, San Diego, CA).

Fiber Photometry Analysis.

Fiber photometry recordings were first combined from the two recording setups (FP3001 and FP3002), and the data structure was matched to allow further processing. Traces were visually inspected and excluded in cases of obvious artifacts or errors due to fiber loosening, bending, etc. Signals were sampled at 20 Hz and aligned to behavioral entry and injection. For each trial, a baseline window was defined from t0 + 5 min to t0 + 28 min. Within this window, we mean-aligned the signal to its isosbestic reference by subtracting the baseline mean difference. We then computed ΔF/F as ($signal_{aligned}$–reference)/reference. The ΔF/F trace was linearly detrended within the analyzed episode and z-scored using the baseline mean and standard deviation to yield ZΔF/F.

For amphetamine recordings, where the signal deviates from baseline due to the amphetamine which precludes detrending, a different approach was applied. A robust linear baseline was estimated within a pre-injection window from 20 to 1 min before injection. To reduce bias from transient elevations, a moving-median envelope (median filter length 10,000 frames ≈ 8.3 min) was computed inside this window and only samples below this envelope were used to fit a linear trend (signal vs. time). The fitted line was then extrapolated across the entire recording and subtracted to remove slow drift (yielding a detrended scaled ΔF/F) The detrended trace was finally z-scored using the mean and standard deviation calculated over the same pre-injection window, producing the z-dF/F outputs used for analysis. For one outlier recording, the effective fit window was shortened within the pre-injection interval before refitting to avoid a misestimated slope.

Global AMPH-induced DA release was quantified as the area under the z-scored response curve (AUC) for each mouse and compared between WT and DAT-I312F/D421N$^{+/+}$ mice.

Analysis of amplitudes of fast DA signals before and after AMPH administration were derived by isolating the fast (~0.01–10 Hz) frequency components of the DA signals during the 30 min baseline recording and 60-min post-injection period. Specifically, the MODWT levels 1–10

(passbands (5–10], (2.5–5], …, (0.0098–0.0195] Hz) were analyzed, collectively covering ~0.01–10 Hz.

Wavelet energy density analysis

Spectral characterization of dLight1.3b signals were analyzed using the maximal overlap discrete wavelet transform (MODWT; mother wavelet 'sym4'). At each level j, the wavelet energy density was computed as the sum of squared coefficients across time and expressed as a percentage of the total energy in the interval. We used the standard dyadic level–frequency bands: $(F_s/2^{j+1}, F_s/2^j]$ Hz, with the maximum resolvable frequency being 10 Hz due to the sampling rate of 20 Hz.

Signal heterogeneity analysis

To compare the heterogeneity of fiber photometry signals, the slow component of the signal was high-pass filtered to exclude the extreme 0.1% at both the upper and lower ends. The remaining signal intensities were segmented into deciles. For each segment, we then calculated the variance of the first-order derivative (9).

Cross-correlation analysis

To examine the temporal coordination of DA dynamics between dorsal and ventral striatum, we performed a cross-correlation analysis. For this we employed a binning and detrending approach, where the signal trace was segmented into consecutive 2-minute bins. Each bin was individually linearly detrended to remove slow drifts or baseline shifts. Cross-correlation functions (xcorr) were then computed separately for each detrended bin. To obtain a representative cross-correlation profile, we averaged the cross-correlation functions from a 20-minute analysis window composed of bins 5 to 14, corresponding to the final 20 minutes prior to vehicle injection.

SUPPLEMENTARY FIGURES:

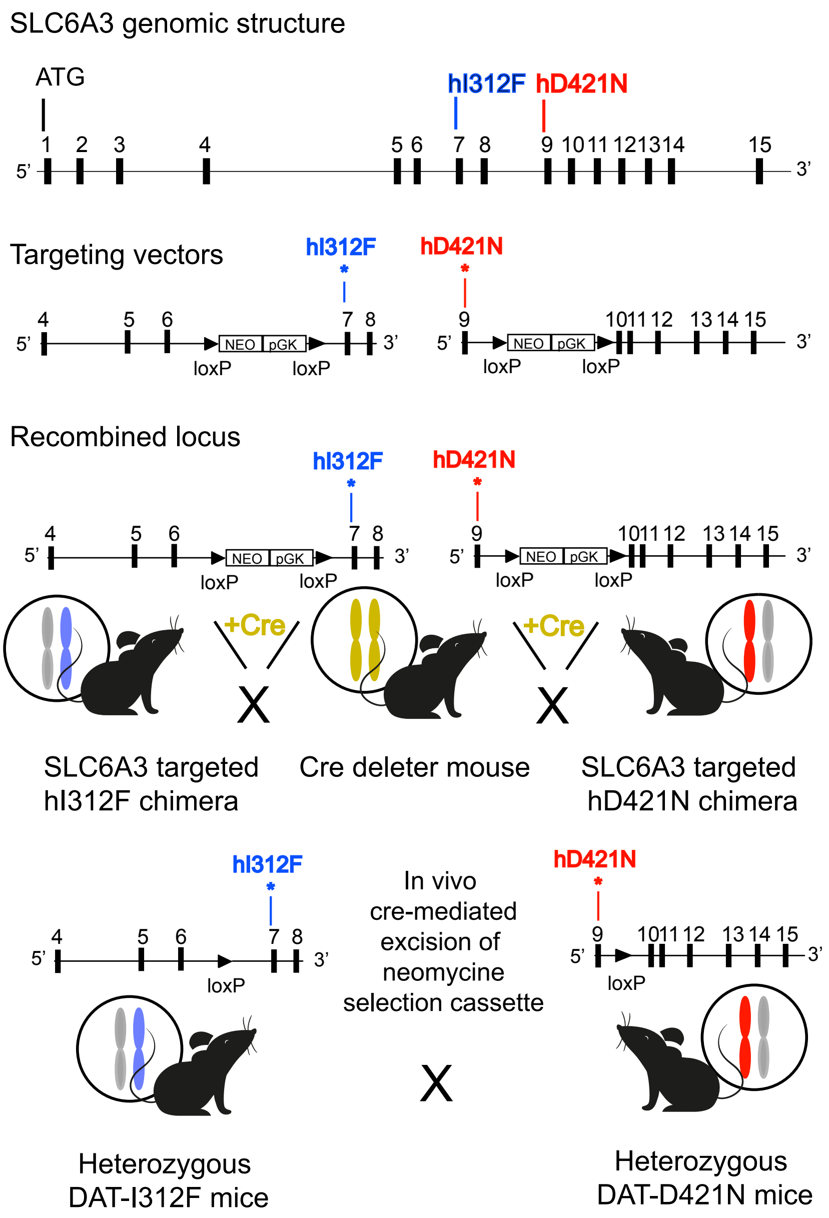

Supplementary Figure 1. Generation of transgenic DAT-I312F/D421N$^{+/+}$ mice. Homologous recombination was used to introduce the hDAT-I312F (mDAT-I311F) and the hDAT-D421N mutation (mDAT-D420N) in exon 7 and 9 of the murine *Slc6a3* gene for expression under the control of the endogenous *Slc6a3* promoter (see Supplementary Methods for further details). The DAT I311F targeting vector contained a murine *Slc6a3* fragment spanning from intron 4 to intron 8 with the point mutation I311F in exon 7 and a neomycin cassette flanked by loxP sites in intron 6. The targeting vector for generation of the DAT-D421N mouse line contained a murine *Slc6a3* region from intron 8 to intron 13 with the D420N point mutation in exon 9 and a loxP-flanked Neomycin cassette in intron 9. Male chimeric mice were bred with Cre-recombinase expressing C57BL/6 mice to excise the *lox*P flanked neomycin cassette. Female DAT-I312F and male DAT-D421N heterozygous mice carrying the neo-excised point mutant knock-in allele were cross-bread to generate compound heterozygous DAT-I312F/D421N$^{+/+}$ mice.

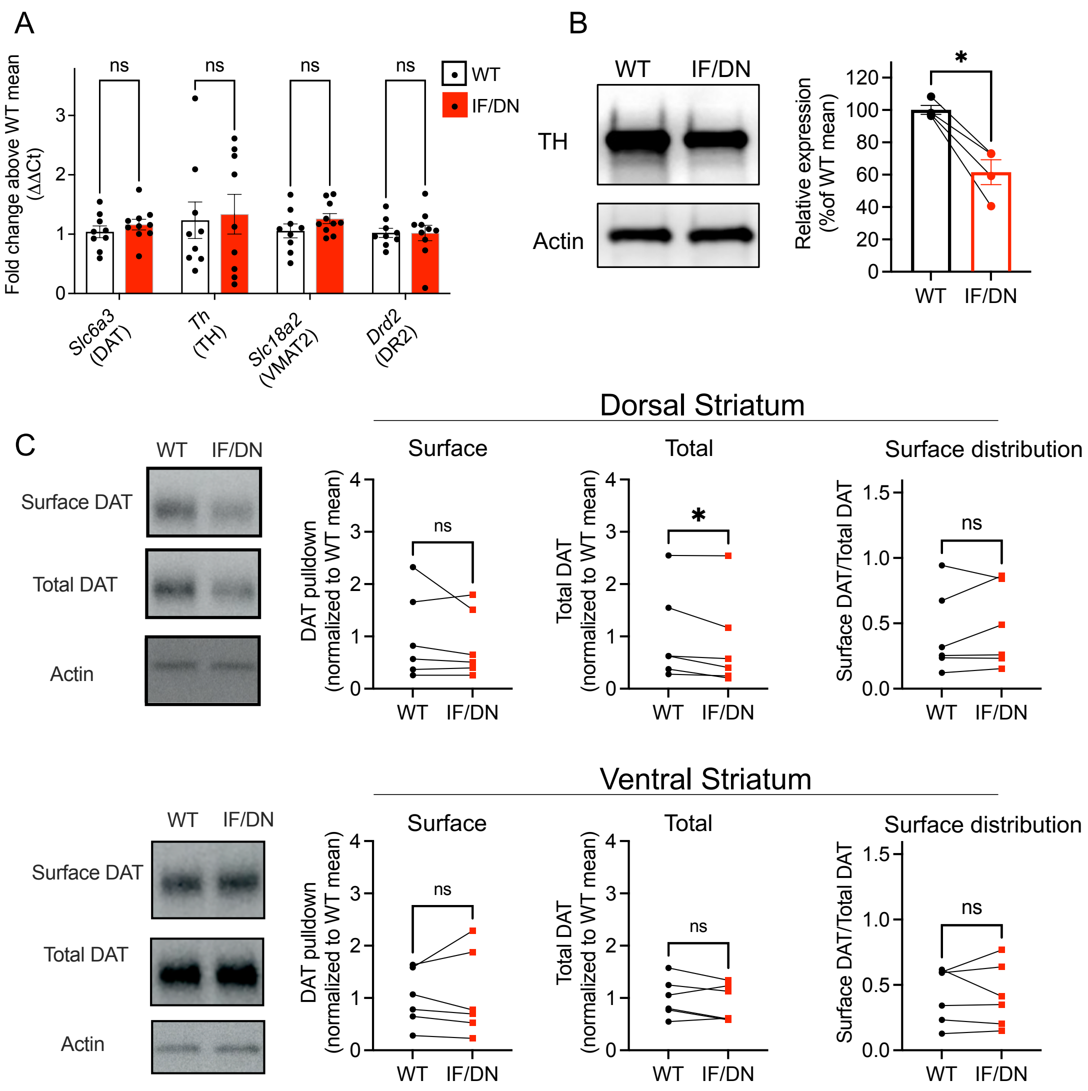

Supplementary Figure 2. Protein and mRNA levels of dopaminergic markers. (A) Midbrain mRNA levels of DAT, TH, VMAT2 and D2R show no genotype differences in transcriptional activity (N=9 WT and 9-10 IF/DN mice, P>0.05, multiple Mann-Whitney tests with Holm-Sidak correction for multiple testing). **(B)** Representative western blot of TH in total striatal lysates with quantification of TH protein band intensities shows a reduction in DAT-I312F/D421N$^{+/+}$ mice relative to WT controls (P<0.05, ratio-paired t-test. N=4 WT:IF/DN pairs, each pair was processed and analyzed in parallel. **(C)** Surface biotinylation of slices from dorsal and ventral striatum. Representative blots are shown in the left panels, followed by average data of biotinylated surface DAT band intensity, total DAT band intensity and quantification of DAT surface distribution, assessed by the DAT surface/total ratio. Whereas the total DAT protein level was significantly reduced in dorsal striatum, no differences in DAT surface distribution were observed between DAT-I312F/D421N$^{+/+}$ and WT mice (ratio-paired t-test N=6 WT:IF/DN pairs, each pair was processed and analyzed in parallel). Data are means ± SEM. *P < 0.05.

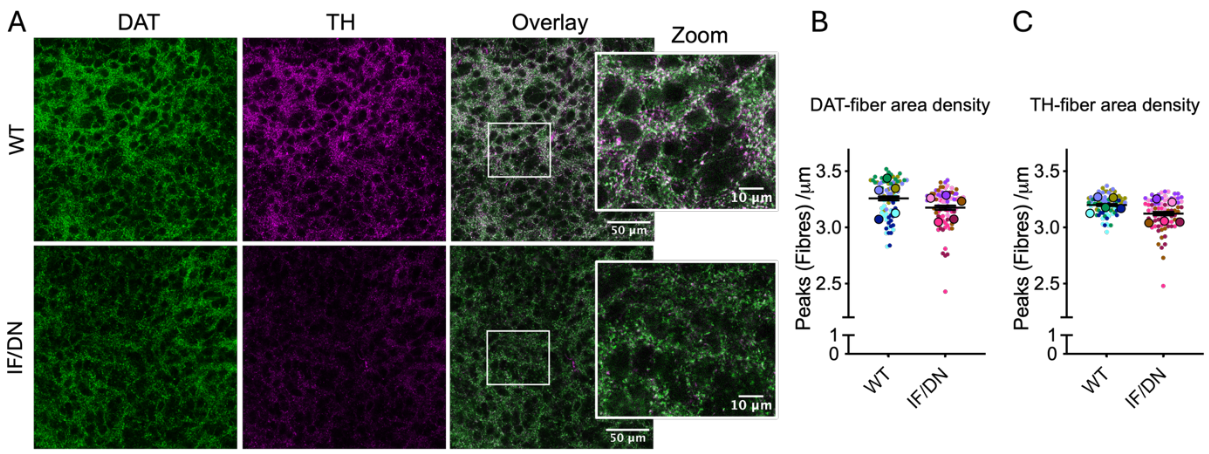

Supplementary Figure 3. DA fiber area density in newborn pups.
(A) Representative confocal images of striatal slices from newborn pups (WT and IF/DN littermates, P1-5) co-labelled for DAT and TH. Images were systematically saquired from striatal slices from 5 WT and 5 IF/DN pups, all stained and imaged in parallel. **(B+C)** Quantification of area density of DAT **(B)** and TH **(C)-**labelled fibers shown as SuperPlots (small points represents images; large points are mouse means). Image level statistic shows significant reductions in both DAT and TH area density ($P < 0.01$ for DAT and $P < 0.001$ for TH, Mann Whitney test, N=80 WT and IF/DN images). Mouse-level statistics: DAT P = 0.31; TH P = 0.22, N=5, Mann-Whitney test). Data are means ± SEM.

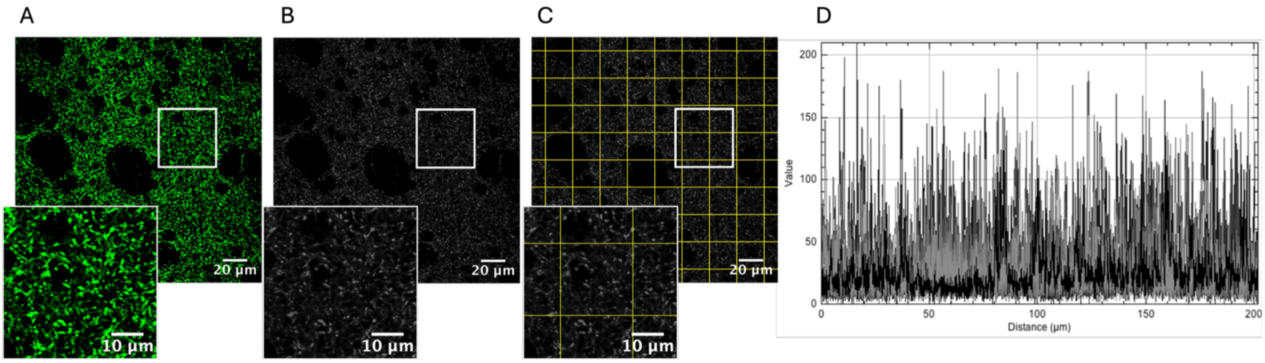

Supplementary Figure 4. Example for the application of Hessian-based edge detection and image intensity profiling in ImageJ. (A) Example image of DAT-labelled dopamine fibers in striatum. **(B)** Application of Hessian-based filter on image in order to extract a second order curvilinear structure from the image. **(C)** Line ROIs drawn, conforming to set grid function in ImageJ. All lines remain in the striatal region. **(D)** ImageJ's "Plot Profile" tool plots the pixel intensities along the length of each line (202.031 µm in length). The given graph displays each line's intensity profile stacked on top of each other. The XY coordinates of the profile can be input to MATLAB software for further analysis.

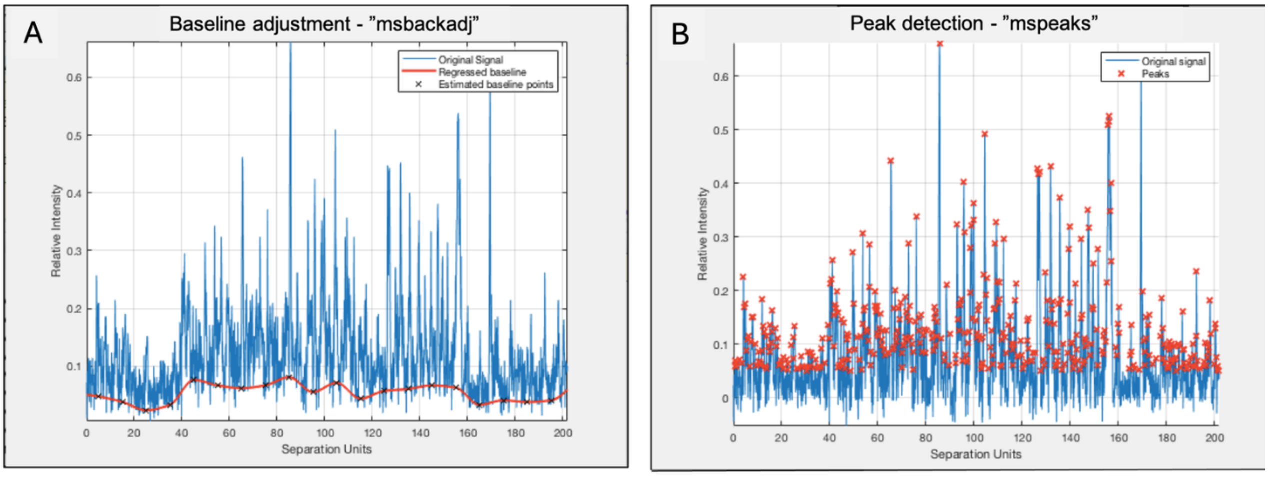

Supplementary Figure 5. Single line scan representative examples for the functions "msbackadj" and "mspeaks". (A) Represented single line scan with baseline displayed after processing data using "msbackadj" function. Intensities have been normalised to the highest intensity detected in the image profiled. The "msbackadj" algorithm classifies the data into signal and background, calculating the average background/threshold in bin sizes of 10 µm (separation units). **(B)** The same represented line scan after baseline adjustment. Detected nerve fibres are marked as Xs (peaks) identified by the "mspeaks" algorithm. The mean of all calculated background values between each WT:IF/DN pair acts as the calculated threshold for the image. All peaks below this threshold, such as those around unit 160 (µm), are not included in the peak detection algorithm. Local maxima are calculated as peaks, representing fibre counts.

SUPPLEMENTARY TABLES

Supplementary Table 1. Kinetic parameters for DA release and reuptake in striatal slices. Kinetic parameters (time to peak (TTP),T1/2, and DA peak) of DA neurotransmission derived from FSCV recordings of single-pulse evoked DA released in dorsal (DS) and ventral striatum (VS) from acute striatal slices. Significant impairments are found across all parameters for the DAT-I312F/D421N$^{+/+}$ mice relative to WT mice (multiple unpaired t-test with Holm-Sidák correction for multiple testing, *p<0.05, **P<0.01 ***P<0.001, ****P<0.0001.). Comparison of the DAT-I312F/D421N$^{+/+}$ genotype penetrance in DS and VS of DAT-I312F/D421N$^{+/+}$ mice was done by normalizing paired recordings from DS and VS to the corresponding means from WT mice. This shows larger impairments in DA release, TTP, and T1/2 in DS than VS (multiple paired t-test with Holm-Sidák correction for multiple testing, #p<0.05, ###P<0.001). Data are shown as means±SEM. N= 9 WT slices and 7 DAT-I312F/D421N$^{+/+}$ mice slices.

	WT			DAT-I312F/D421N$^{+/+}$			DAT-I312F/D421N$^{+/+}$ % of corresponding WT region		
	Striatum total	DS	VS	Striatum total	DS	VS	Striatum total	DS	VS
**TTP (s)**	0.24±0.02	0.24 ±0.02	0.28±0.02	0.66±0.06 ****	0.67 ± 0.09 **	0.61±0.05 ***	240±30 ***	280 ± 50 **	210±20 ***/#
**T1/2 (s)**	0.42 ±0.02	0.42 ±0.03	0.46 ±0.02	2.2 ±0.2 ****	2.4 ± 0.3 ***	2.1 ±0.2 ***	500 ±50 ****	580 ± 80 **	460 ±50 ***/#
**DA peak (µM)**	1.0±0.05	1.00±0.08	0.95±0.05	0.16±0.02 ****	0.060±0.02 ****	0.22±0.03 ****	16±3 ****	6.0±2 ****	23±3 ****/###
***significantly different from corresponding region in WT mice** **#significantly different from DS**									

Supplementary Table 2. stereological counting of TH-positive neurons in Substantia nigra: Precision of cell number and volume estimates

	Cell number estimations				Volume estimations			
	Section sampling fraction	Number of sections	Number of counts	CE	Section sampling fraction	Number of sections	Number of counts	CE
**WT**	1/4	8 [7-9]	199.22 [111-254]	0.07	1/4	8 [7-9]	611.4 [374-898]	0.011
**DAT – I312F/D421N**	1/4	8.6 [7-9]	208.75 [150 - 299]	0.069	1/4	8.6 [7-9]	593.5 [387-789]	0.011
**Values are mean [range], CE = coeficient of error**								

Supplementary Table 3. Stereological counting of TH-positive neurons in Substantia nigra: Total cell number and volume

	Total cell number		Volume cm^3	
	Total TH-positive cells	CV	Volume	CV
WT	7981 [6081-10054]	0.14	0.573 [0.44-0.76]	0.16
DAT-I312F/D421N$^{+/+}$	7297 [5091.2-9801.6]	0.18	0.622 [0.54-0.68]	0.09
Unpaird t-test with Welch's correction	P=0.275		P=0.421	
Values are mean [range], CV = coeficient of variation				

Supplementary Table 4. Primary antibodies

Target	species	supplier	Applied concentration
TH	rabbit	Millipore (Cat#AB152, RRID:AB_390204)	1:90000 (IHC, stereology)
TH	rabbit	Thermo Fisher Scientific (Cat#OPA1-04050, RRID:AB_325653)	1:1000 (IHC, WB)
TH	mouse	Millipore (Cat#MAB318, RRID:AB_2201528)	1:1000 (IHC, WB)
DAT	rat	Millipore (Cat#MAB369, RRID:AB_2190413)	1:1000 (IHC, WB)
VMAT2	rabbit	kindly provided from Gary Miller's Lab	1:1000 (IHC)<br>1:2000 (WB)
DR1	rat	Sigma Aldrich (Cat#D2944, RRID:AB_1840787)	1:500 (IHC)<br>1:1000 (WB)
DR2	rabbit	Synaptic Systems (Cat#376 203, RRID:AB_2636918)	1:500 (IHC)<br>1:1000 (WB)
Alpha-synuclein	mouse	BD Transduction Labrotatories (Cat#610787, RRID:AB_398108)	1:1000 (WB)
Phospho-a-synuclein (ser129)	rabbit	Cell Signaling Technology (Cat#23706, RRID AB_2798868)	1:1000 (WB)

HRP-conjugated anti–β-actin antibody	mouse	Sigma Aldrich (Cat#A3854, RRID:AB_262011)	1:40000
HRP-conjugated anti-rat	goat	Thermo Fisher Scientific (Pierce) (Cat#31470, RRID:AB_228356)	1:2000
HRP-conjugated anti-mouse	goat	Thermo Fisher Scientific (Cat#31430, RRID:AB_228307)	1:2000
HRP-conjugated anti-rabbit	goat	Cell Signaling (Cat#7074S, RRID:AB_2099233)	1:2000
Alexa Fluor 488 anti rat	goat	Thermo Fisher Scientific Cat#A-11006, RRID:AB_2534074	1:400
Alexa Fluor 568 anti rabbit	goat	Thermo Fisher Scientific Cat#A-11036, RRID:AB_10563566	1:400
Alexa Fluor 568 anti mouse	goat	Thermo Fisher Scientific Cat#A-11031, RRID:AB_144696	1:400
Alexa Fluor 647 anti rabbit	goat	Thermo Fisher Scientific Cat#A-21245, RRID:AB_2535813	1:400

Supplementary Table 5. qPCR primers

Target Gene	Primer	Primer Sequence
Drd1	Forward	TCTCCCAGATCGGGCATT
	Reverse	GTCACTTTTCGGGGATGCTG
Drd2	Forward	TCGTCACCCTGCTGGTCTAT
	Reverse	TGGGTACAGTTGCCCTTGAGT
TH	Forward	CCCCACCTGGAGTATTTTGTG
	Reverse	ATCACGGGCGGACAGTAGACC
SLC18A2	Forward	GGTATGCTATCGGTCCCTCT
	Reverse	AGCCAAGGAAAGCCAAT
SLC6A3	Forward	TGCTCTACTTCAGCCTGTGG
	Reverse	TATGCTCTGATGCCATCCAT
Gapdh	Forward	AAGGGCTCATGACCACAGTC
	Reverse	GGATGCAGGGATGATGTTCT
HPRT	Forward	CAGCGTCGTGATTAGCG
	Reverse	GCCTCCCATCTCCTTCAT

SUPPLEMENTARY MOVIES

Supplementary movies 1-4. Show example video recordings of WT (movie 1+3) and DAT-I312F/D421N$^{+/+}$ mice (movie 2+4) 30 min (movie 1+2) and 120 min (movie 3+4) into an OFT.

Supplementary movies 5-6.

Recordings of clasping behavior during tail suspension shown at 50% speed. Supplementary movie 5 displays a WT mouse and supplementary movie 6 a DAT-I312F/D421N$^{+/+}$ mouse with hindlimb clasping.

REFERENCES

1. Stewart A, Mayer FP, Gowrishankar R, Davis GL, Areal LB, Gresch PJ, et al. Behaviorally penetrant, anomalous dopamine efflux exposes sex and circuit dependent regulation of dopamine transporters. *Mol Psychiatry.* 2022;27(12):4869-80.
2. Mayer FP, Stewart A, Varman DR, Moritz AE, Foster JD, Owens AW, et al. Kappa Opioid Receptor Antagonism Restores Phosphorylation, Trafficking and Behavior induced by a Disease Associated Dopamine Transporter Variant. *bioRxiv.* 2024.
3. Sathyanesan A, Ogura T, and Lin W. Automated measurement of nerve fiber density using line intensity scan analysis. *J Neurosci Methods.* 2012;206(2):165-75.
4. Meijering E. FeatureJ: An ImageJ Plugin Suite for Image Feature Extraction. http://imagescience.org/meijering/software/featurej.
5. Utsunomiya S-i, Fujita Y, Tanaka S, Kajihara S, Aoshima K, Oda Y, et al. Signal Processing Algorithm Development for Mass++ (Ver. 2): Platform Software for Mass Spectrometry. *IPSJ Transactions on Bioinformatics.* 2014;7(0):24-9.
6. Gundersen HJ, and Jensen EB. The efficiency of systematic sampling in stereology and its prediction. *J Microsc.* 1987;147(Pt 3):229-63.
7. Gundersen HJ, Jensen EB, Kieu K, and Nielsen J. The efficiency of systematic sampling in stereology--reconsidered. *J Microsc.* 1999;193(Pt 3):199-211.
8. Huang D, Wang Z, Tong J, Wang M, Wang J, Xu J, et al. Long-term Changes in the Nigrostriatal Pathway in the MPTP Mouse Model of Parkinson's Disease. *Neuroscience.* 2018;369:303-13.
9. Jorgensen SH, Ejdrup AL, Lycas MD, Posselt LP, Madsen KL, Tian L, et al. Behavioral encoding across timescales by region-specific dopamine dynamics. *Proc Natl Acad Sci U S A.* 2023;120(7):e2215230120.
10. Lopes G, Bonacchi N, Frazao J, Neto JP, Atallah BV, Soares S, et al. Bonsai: an event-based framework for processing and controlling data streams. *Front Neuroinform.* 2015;9:7.
