# Supplementary figures and images for: Mouse model of atypical DAT deficiency syndrome uncovers dopamine dysfunction associated with parkinsonism and ADHD
Source: J Clin Invest. 2026 Jan 27;136(6):e169297. doi: 10.1172/JCI169297 (PMC12987629; doi:10.1172/JCI169297)

Figure 2C

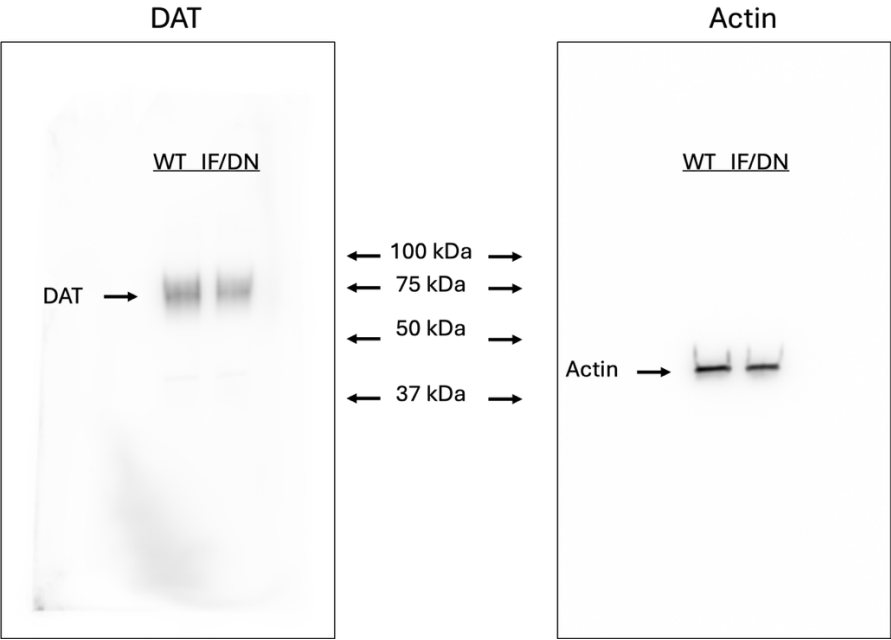

Figure 2F

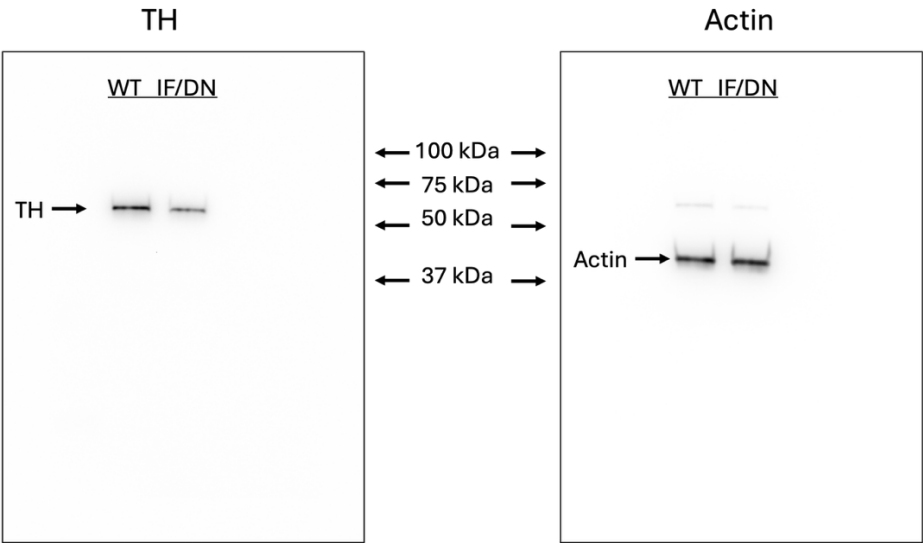

Figure 2I

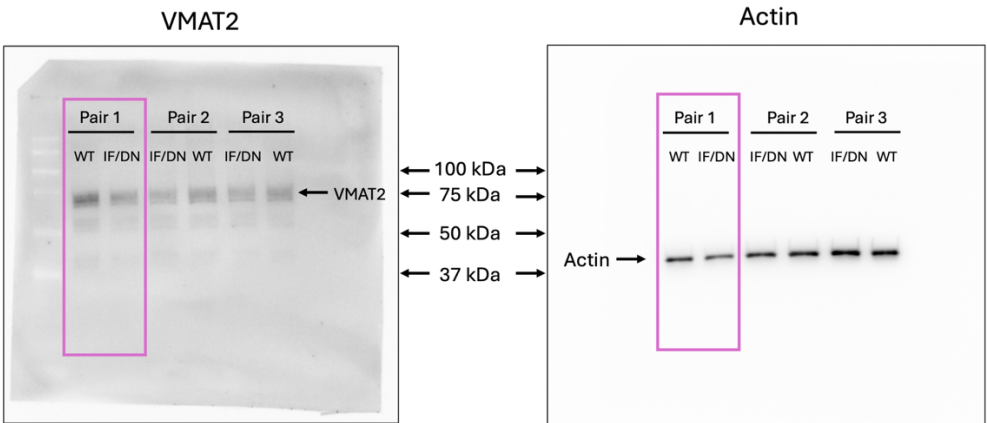

**Figure 3E**

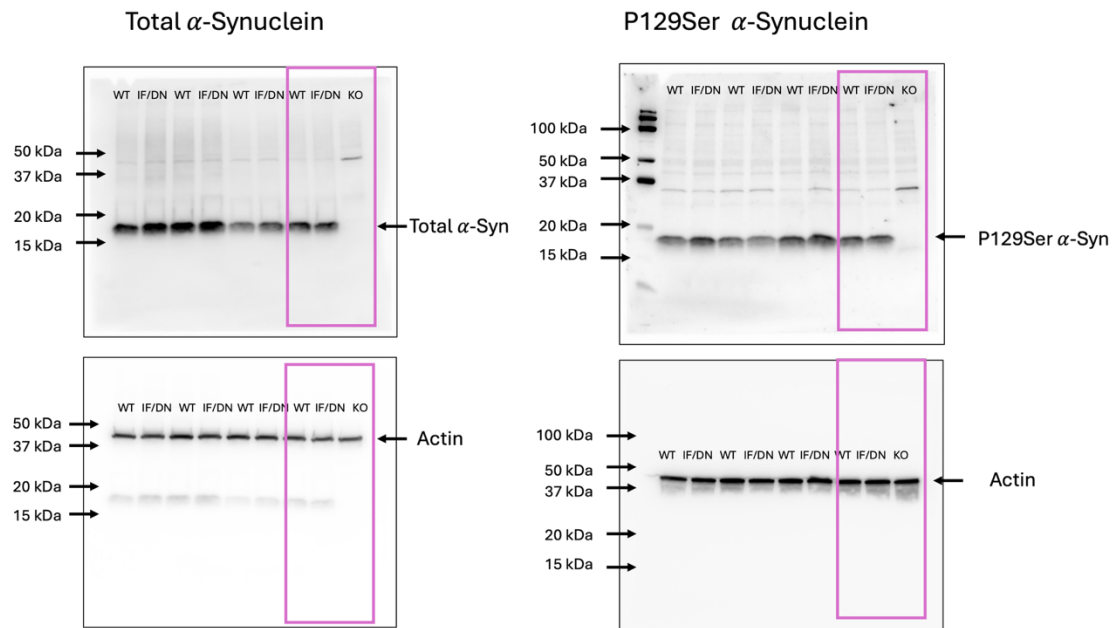

**Figure 4B+E**

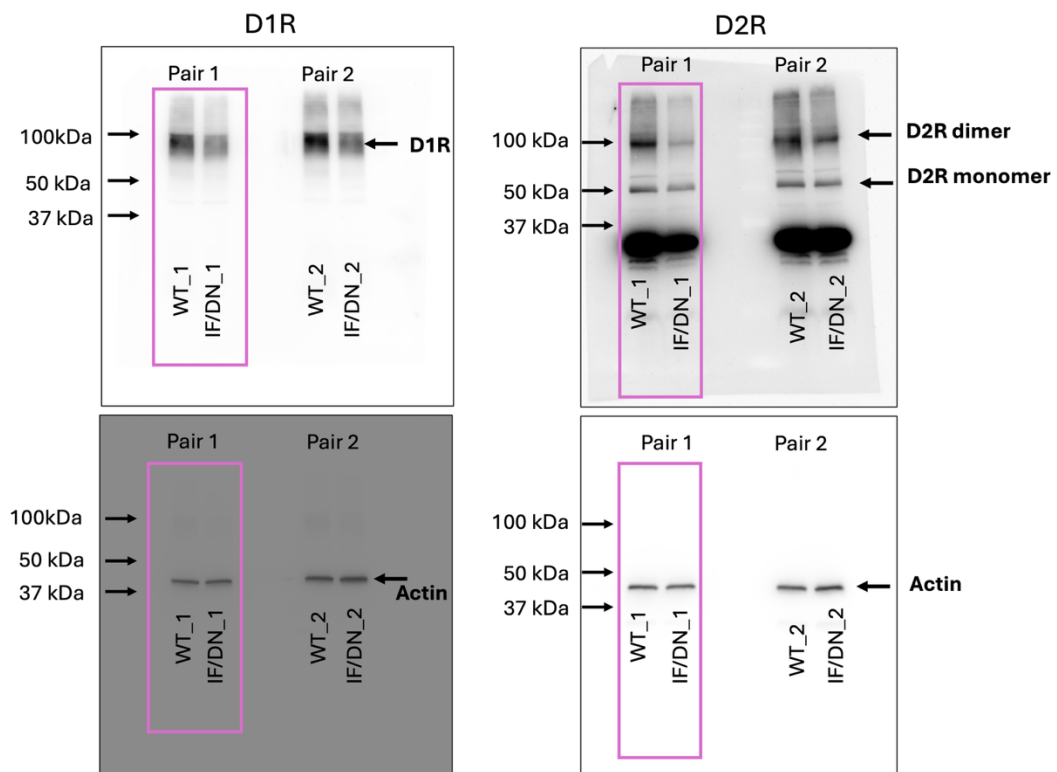

**Supplementary Figure 2B**

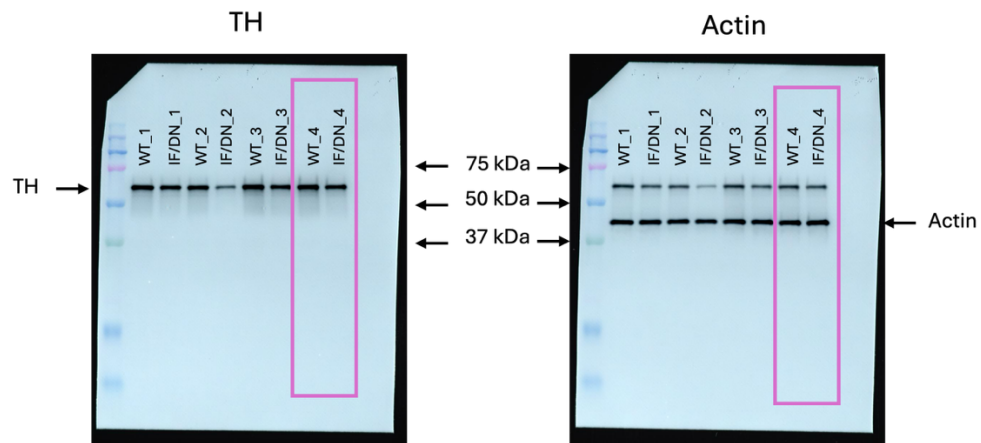

**Supplementary Figure 2C**

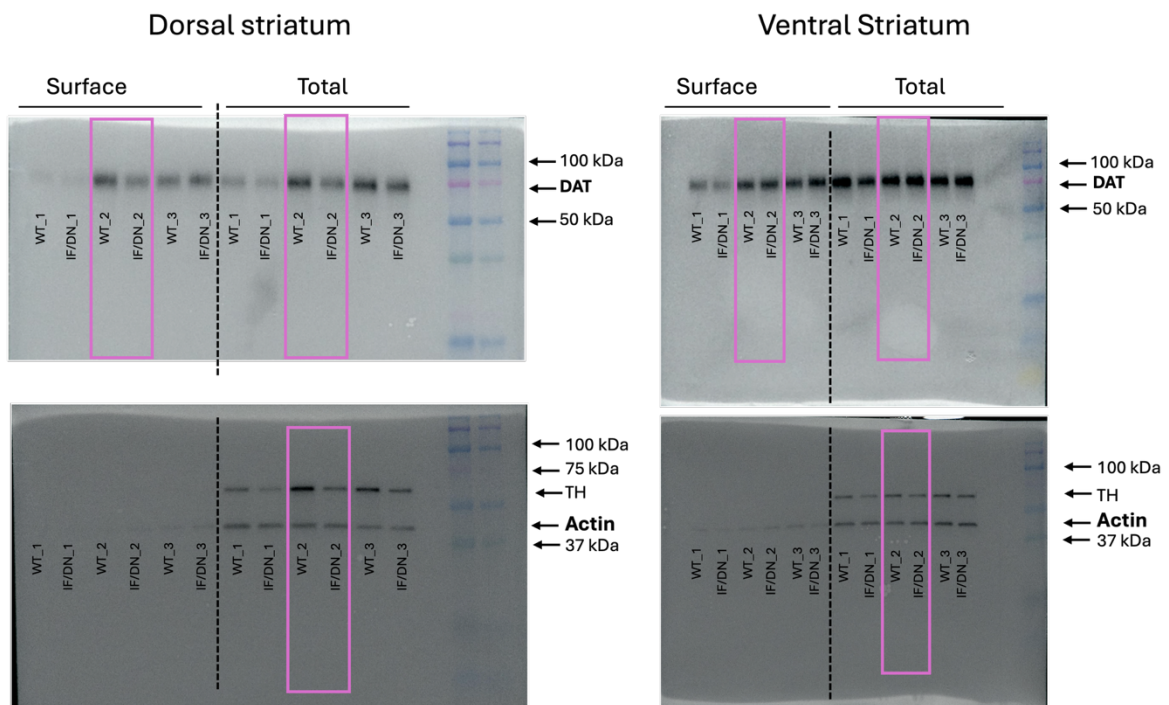

Supplement: Unedited blot and gel images [file jci-136-169297-s042.pdf]
